# Microbiota-derived indoles alleviate intestinal inflammation and modulate microbiome by microbial cross-feeding

**DOI:** 10.1186/s40168-024-01750-y

**Published:** 2024-03-19

**Authors:** Gang Wang, Yuxin Fan, Guolong Zhang, Shuang Cai, Yonghang Ma, Lijie Yang, Yuming Wang, Haitao Yu, Shiyan Qiao, Xiangfang Zeng

**Affiliations:** 1 State Key Laboratory of Animal Nutrition, College of Animal Science and Technology, China Agricultural University, Beijing, 100193 China; 2 Beijing Key Laboratory of Biological Feed Additive, China Agricultural University, Beijing, 100193 China; 3 Frontier Technology Research Institute of China Agricultural University in Shenzhen, Shenzhen, 518116 China; 4 Department of Animal and Food Sciences, Oklahoma State University, Stillwater, OK 74078 USA

**Keywords:** Microbial tryptophan metabolites, Indole derivatives, Intestinal inflammation, *Lactobacillus*

## Abstract

**Background:**

The host–microbiota interaction plays a crucial role in maintaining homeostasis and disease susceptibility, and microbial tryptophan metabolites are potent modulators of host physiology. However, whether and how these metabolites mediate host–microbiota interactions, particularly in terms of inter-microbial communication, remains unclear.

**Results:**

Here, we have demonstrated that indole-3-lactic acid (ILA) is a key molecule produced by *Lactobacillus* in protecting against intestinal inflammation and correcting microbial dysbiosis. Specifically, *Lactobacillus* metabolizes tryptophan into ILA, thereby augmenting the expression of key bacterial enzymes implicated in tryptophan metabolism, leading to the synthesis of other indole derivatives including indole-3-propionic acid (IPA) and indole-3-acetic acid (IAA). Notably, ILA, IPA, and IAA possess the ability to mitigate intestinal inflammation and modulate the gut microbiota in both DSS-induced and IL-10^−/−^ spontaneous colitis models. ILA increases the abundance of tryptophan-metabolizing bacteria (e.g., *Clostridium*), as well as the mRNA expression of acyl-CoA dehydrogenase and indolelactate dehydrogenase in vivo and in vitro, resulting in an augmented production of IPA and IAA. Furthermore, a mutant strain of *Lactobacillus* fails to protect against inflammation and producing other derivatives. ILA-mediated microbial cross-feeding was microbiota-dependent and specifically enhanced indole derivatives production under conditions of dysbiosis induced by *Citrobacter rodentium* or DSS, but not of microbiota disruption with antibiotics.

**Conclusion:**

Taken together, we highlight mechanisms by which microbiome-host crosstalk cooperatively control intestinal homoeostasis through microbiota-derived indoles mediating the inter-microbial communication. These findings may contribute to the development of microbiota-derived metabolites or targeted “postbiotic” as potential interventions for the treatment or prevention of dysbiosis-driven diseases.

Video Abstract

**Supplementary Information:**

The online version contains supplementary material available at 10.1186/s40168-024-01750-y.

## Background

Intestinal dysbiosis characterized by microbiota imbalance is associated with inflammation, oxidative stress and a variety of diseases including metabolic disorders and inflammatory bowel disease (IBD). Metabolites secreted or modified by the microbiota or host mediate host–microbiota interactions. They constitute a complicated network and co-together maintain the functions of the host, the microbiota and their interdependence. Fecal microbiota transplantation (FMT) has emerged a promising therapy for dysbiosis-driven diseases [1], and strategies to restore gut microbiota homeostasis may have potential to alleviate these conditions. It has also been reported that transferring the microbiota of inflamed individuals to wild-type, germ-free receptors increases their susceptibility to colitis [2]. Although metabolites are involved in regulating the host immune system as signaling molecules and affect the efficacy of cancer chemotherapy and host metabolism through activating relevant signaling pathways [3–5], the mechanisms by which these small molecules regulate the intestinal microbiome remain poorly understood.

Tryptophan is an essential amino acid for protein synthesis and a precursor to various metabolites that are crucial for host metabolism and physiology [6]. Tryptophan can be metabolized by the host via the kynurenine or serotonin (5-HT) pathways or by the gut microbiota via the indole pathway [5]. There is growing evidence that indole derivatives metabolized by various microbiota such as indole-3-lactate (ILA), indole-3-propionic acid (IPA), and indole-3-acetate (IAA), have a profound impact on host immunity and intestinal function [2, 5]. These microbiota-derived tryptophan metabolites have been shown to strengthen the intestinal barrier, protect against pathogens, and influence host metabolism by binding to the aryl hydrocarbon receptor (AhR) or pregnane X receptor (PXR) [2, 7–10]. Some probiotics regulate intestinal homeostasis through indole derivative-induced AhR activation. For example, *Lactobacillus reuteri* promotes the differentiation and expansion of oral tolerogenic CD4^+^CD8αα^+^ intraepithelial T lymphocytes through the production of ILA [11], which is capable of inhibiting the polarization of Th17 cells in vitro [12]. Additionally, *Bifidobacterium* in breastmilk secretes ILA and helps regulate immune system maturation in early life [13].

The recovery of dysbiotic microbiota or amelioration of associated disease is usually accompanied by an increase of several special metabolites, such as short-chain acids (SCFAs) and indole derivatives [14, 15], yet whether it is the direct effect of these increased metabolites produced in vivo still unknown. Whether and how these metabolites exert inter-bacterial signaling activities to regulate the microbiome likewise needs to be elucidated. In the present study, we chose *Lactobacillus reuteri*, a probiotic with the ability to modulate intestinal immunity and gut microbiota, to investigate the role and mechanisms of indoles in mediating host–microbiota interactions and inter-microbial communication. We found that ILA is a key molecule of *Lactobacillus* in the alleviation of inflammation and modulation of the gut microbiota. ILA increased the expression of relevant enzymes involved tryptophan metabolism via bacterial cross-feeding and promoted the synthesis of microbial tryptophan metabolites including IPA and IAA in vivo and in vitro. The regulation of the intestinal microbiota by ILA was microbiota-dependent and specifically increasing indoles levels under conditions of dybiosis induced by *Citrobacter rodentium* or DSS, but not of microbiota disruption with antibiotics. The anti-inflammatory and intestinal barrier-strengthening effects of IPA were directly regulated by PXR activation, while those of IAA were microbiota-dependent. Together, these results collectively suggested ILA-mediated the inter-microbial communication modulates the microbiome-host crosstalk and controls intestinal homeostasis in conjunction with other microbiota-derived indoles.

## Methods

### Animal studies and ethical statement

C57BL/6 mice (6–8 weeks old) were obtained from Beijing HFK Bioscience, while *IL-10*^−*/*−^ mice (6–8 weeks old) were purchased from Guangzhou Cyagen Biosciences. All mice were housed under a 12-h light/dark cycle and fed a standard chow diet and water ad libitum.

### Bacterial strains and preparation

*L. reuteri* I5007 was isolated from the colonic mucosa of healthy weaning piglets and further confirmed through whole-genome sequencing (NCBI ID: 1340495); this strain was found to be chloramphenicol, kanamycin, and streptomycin-resistant. *L. reuteri* I5007, mutant *L. reuteri* I5007, *L. johnsonii*, *L. plantarum*, *L. delbrueckii*, and *L. rhamnosus* GG were cultured in MRS medium (Solarbio Science and Technology Co., Beijing, China) at 37 ℃ for 20 h under anaerobic conditions. Bacterial cells were collected by centrifugation at 12,000 rpm for 15 min. Heat-killed *L. reuteri* I5007 was prepared by heating at 95 °C for 20 min. No colonies were detected after 48 h of culture at 37 °C after heat treatment. *Clostridium sporogenes* (ATCC 15579; American Type Culture Collection) was cultured in fluid thioglycolate medium in an anaerobic atmosphere at 37 °C for 24 h. *C. rodentium* (ATCC 51459; American Type Culture Collection) was inoculated into LB broth and cultured in a shaker overnight at 37 °C. Overnight culture was then centrifuged at 12,000 rpm for 15 min, washed once, and resuspended at 10^8^ CFU/mL in PBS. *L. reuteri* I5007 was cultured anaerobically with or without tryptophan at 0.01, 0.1, or 1 mM at 37 °C for 20 h prior to measurement of indole derivatives in the supernatant.

### Homologous recombination

ArAT-deficient strain of *L. reuteri* I5007 was constructed by homologous recombination as described previously [16]. Briefly, 871-bp and 850-bp DNA fragments were separately cloned from the upstream and downstream sequences of the ArAT gene of *L. reuteri* I5007 and then fused with an erythromycin resistance gene fragment amplified from the pMG36e plasmid. Recombinant DNA was cloned into the pLCNICK-2179 plasmid using TA cloning. The resulting plasmid was inserted into competent *L. reuteri* I5007 through electroporation, followed by plating onto the MRS medium agar plates containing erythromycin. An ArAT-knockout strain was obtained and confirmed for the absence of the ArAT gene by PCR of bacterial genomic DNA.

### Colonic microbiota transplantation

Fresh donor colonic contents were pooled and homogenized 5 times and diluted with sterile saline containing 20% (v/v) glycerol. The pooled samples were centrifuged at 500 × *g* for 3 min at 4 °C, and the supernatant was collected and stored at − 80 °C. Prior to colonic microbiota transplantation (CMT), glycerol in each sample was replaced with PBS by centrifugation and the number of live microbial cells was counted using methylene blue staining [16].

### DSS-induced colitis

C57BL/6 mice were given 3% (wt/vol) DSS (36,000–50,000 Da, MP Biomedicals) in drinking water for 7 days. Water was changed every 2 days. The animals were weighed daily for the entire duration. The DAI was evaluated to assess the severity of colitis as previously described [17].

### Antibiotic treatment

Mice were given an antibiotic cocktail of ciprofloxacin (0.2 g/L), vancomycin (0.5 g/L), and metronidazole (1 g/L) [18] in drinking water for 2 weeks prior to other treatments. Water was replenished every week.

### Intestinal permeability assessment

Fluorescein isothiocyanate (FITC, 4 kDa, Sigma)-dextran was dissolved in PBS at 100 mg/ml. After 6 h of fasting, mice were orally administered with 600 mg/kg FITC-dextran. Blood was collected after 4 h and serum was prepared. The fluorescence of each serum sample was measured at an excitation wavelength of 490 nm and an emission wavelength of 530 nm.

### Histological analysis and goblet cell evaluation

The colon tissue was rolled, fixed with 4% paraformaldehyde for 24 h, embedded in paraffin wax, sliced (5 μm), and stained with hematoxylin and eosin (H&E) or alcian blue. The pathological analysis of H&E-stained sections was scored blindly by two independent investigators. The scoring system was as follows: ulcer size (0 = none, 1 = less than 3 mm, 2 = more than 3 mm), severity of inflammation (0 = none, 1 = mild, 2 = moderate, and 3 = severe), fibrotic degree (0 = none, 1 = mild, 2 = moderate, and 3 = severe), extent of epithelial/crypt damage (0 = none, 1 = basal 1/3, 2 = basal 2/3, 3 = crypt loss, 4 = crypt and surface epithelial destruction), and injury depth (0 = none, 1 = mucous layer, 2 = submucosa, 3 = muscle layer, 4 = serous layer).

### Anaerobic culture of the microbiota

Cell-free supernatant (CFS) of *L. reuteri* was obtained by centrifugation of the bacterial culture at 12,000 rpm for 15 min, followed by filtration through a 0.22-µm filter and dilution to 10% with the corresponding culture. For in vitro anaerobic culture of colonic microbiota, approximately 100 mg of the colonic content was vortexed in 5 ml BHI medium under anaerobic conditions. MRS medium (200 µl), CFS (10%), or ILA (1 mM) was then added to the bacterial culture and anaerobically incubated for 12 h at 37 °C in a GasPak EZ Anaerobe Pouch system. *C. sporogenes* was co-cultured with or without an addition of tryptophan (0.3g/L), the strain and CFS of *L. reuteri* I5007 or ArAT-deficient mutant *L. reuteri* I5007 (△L.R), ILA for 24 h at 37 °C in an anaerobic atmosphere.

### Isolation of lymphocytes and flow cytometry

The mesenteric lymph nodes (MLNs) were collected and prepared as single-cell suspensions. The MLNs were then passed through a 70-μm filter and resuspended in FACS buffer for flow cytometric analyses. Common lymphoid progenitor (CLP) cells were isolated as previously described [19]. Briefly, the colon was opened up longitudinally and washed to remove the contents by scraping gently with PBS. The tissue were then diced into small pieces and incubated in prewarmed sterile HBSS (without Ca^2+^ and Mg^2+^) containing 3% fetal bovine serum (FBS), 10 mM EDTA, and 5 mM dithiothreitol (DTT) for 30 min at 37°C in a shaking water bath. The cells, including immune cells and enterocytes, were recovered by filtration through a 100-μm filter. Cells were then dissociated from each other in prewarmed HBSS (with Ca^2+^ and Mg^2+^) containing 3% FBS, 1% L-glutamine, 1% penicillin–streptomycin, 10 mM HEPES, 0.5 mg/mL collagenase D, 0.5 mg/mL Dispase, and 0.04 mg/mL DNase I for 45 min at 37 °C while shaking. Cell suspensions were filtered through a 70-µm cell strainer into precooled sterile PBS, followed by centrifugation in a 40/80 Percoll gradient for 20 min at 1000 × *g* at 37°C. CLPs were obtained by collecting the cells at the interface and washing twice in FACS buffer for flow cytometric analysis.

For detection of Th1, Th2, Th17F, and Th17A cells, lymphocytes were stimulated for 4 h with 50 ng/mL PMA (Sigma) and 1 µg/mL ionomycin (Sigma) in the presence of GolgiStop (BD Biosciences). Cells were first stained for surface CD4 and then fixed and permeabilized with a BD Cytofix/Cytoperm Kit, followed by intracellular staining with the following antibodies: PerCP/Cy5.5-IL-4, FITC-IFN-γ, AF647-IL-17F, and PE/Cy7-IL-17A. To quantify Treg cells, a True-Nuclear Transcription Factor Buffer Set (BioLegend) was used to label cells with anti-CD4-PE, anti-CD25-FITC, and anti-FoxP3-Alexa Fluor. The percentage of each T cell type was measured with BD FACS Celesta and FlowJo analysis software (BD Biosciences). Compensation was set using UltraComp eBeads.

### Western blot analysis

The colonic tissues were lysed with RIPA extraction buffer (Solarbio, China) containing a 1% protease inhibitor cocktail. Following centrifugation at 12,000 rpm for 15 min at 4°C, the supernatant was collected and the total protein concentration was quantified using a bicinchoninic acid (BCA) protein assay kit (Solarbio, China). Proteins were separated by SDS–polyacrylamide gel electrophoresis and transferred onto polyvinylidene difluoride (PVDF) membranes (Millipore, USA). The membranes were blocked with 5% skim milk for 1.5 h and incubated with primary antibodies against PXR (Abcam, USA), E-cadherin (Cell Signaling Technology, USA), Cyp1a1 (Cell Signaling Technology, USA), and occludin (Cell Signaling Technology, USA) overnight at 4°C. The membranes were washed and incubated with HRP-labeled secondary antibodies at room temperature for 2 h, followed by signal detection with an ECL Kit (Solarbio, China).

### RNA extraction and quantitative PCR

Total RNA from cells and tissue was extracted using TRIzol reagent (Takara, Japan). The concentration of RNA was determined with NanoDrop 2000 (Thermo Fisher Scientific, Wilmington, DE, USA). Quantitative PCR was performed as previously described [20] using gene-specific primers and glyceraldehyde-3-phosphate dehydrogenase (GAPDH) as the reference gene (Additional file 2: Table S2). The relative mRNA expression level of each target gene was determined using the 2^−△△Ct^ method.

### ELISA

Inflammatory cytokines in the serum and colonic tissue samples were measured with enzyme-linked immunosorbent assay (ELISA) kits (Cusabio, Baltimore, MD, USA) according to the manufacturer’s instructions.

### Determination of tryptophan metabolites

The colonic contents (20 mg) were homogenized in 200 µl water and mixed with 600 µl of precooled methanol containing internal standard (1-methyl-tryptophan, 2.0 µg/ml). Serum and CFS samples (30 µL) were mixed with 120 µl methanol containing 1-methyl-tryptophan. The mixture was then vortexed for 5 min and centrifuged at 16,000 rpm for 10 min at 4℃. The supernatant was vacuum-dried and reconstituted with 100 µl of 10% methanol and centrifuged at 16,000 rpm for 15 min at 4℃ prior to the UHPLC–MS/MS analysis. An Agilent 1290 Infinity II UHPLC coupled to an Agilent 6470A triple quadrupole (QQQ) mass spectrometer was used for tryptophan metabolite analysis. The mobile phase consisted of 0.1% formic acid in water (A) and methanol (B). Chromatographic separation was conducted using a gradient elution program for the detection of tryptophan metabolites as follows: 0 min, 95% B; 2 min, 95% B; 8 min, 50% B; 10 min, 5.0% B; 12 min, 5.0% B; 13 min, 95% B; 16 min, 95% B. The gradient elution program for the detection of AAAs was as follows: 0 min, 95% B; 2 min, 95% B; 6 min, 50% B; 7.5 min, 5.0% B; 9 min, 5.0% B; 10 min, 95% B; and 15 min, 95% B. The column temperature was 35 °C, and the flow rate was 0.3 ml/min. For analysis, the eluate from the column was ionized by an electrospray ionization source in positive mode (ESI +) under the following settings: gas temperature: 300°C, gas flow: 5 L/min, nebulizer: 45 psi, ShenthGasHeater: 300 °C, SheathGasFlow: 10 L/min, and capillary voltage: 4000 V. Dynamic multiple reaction monitoring (dMRM) was used for quantitative analysis. Data were acquired and processed in Agilent MassHunter software (version B.04.00).

### DNA extraction, 16S rRNA gene sequencing, and data analysis

Total microbial DNA was extracted from the colonic contents using the QIAamp DNA Stool Mini Kit (Qiagen, Hilden, Germany) according to the manufacturer’s instructions. Extracted DNA was quantified using a NanoDrop 2000 (Thermo Fisher Scientific, Wilmington, DE, USA). The V3–V4 hypervariable region of the 16S rRNA gene was amplified using universal primers 338F (5′-GTGCCAGCMGCCGCGG-3′) and 806R (5′-CCGTCAATTCMTTTRAGTTT-3′). Agarose gel electrophoresis was performed to confirm the size of PCR products. Amplicons were quantified, pooled, and sequenced on the Illumina MiSeq system (Illumina, San Diego, CA, USA) for paired-end reads. Sequence analysis was performed using the UNOISE pipeline through USEARCH v10.0 [21] and VSEARCH v2.15 [22]. Briefly, paired-end sequences were merged, quality filtered, and decomplexed using VSEARCH. Denoising and chimera removal were conducted using UNOISE3 to correct for sequencing errors. Assembled sequences were mapped back to the denoised chimera-free sequences as OTUs with 97% identity. The taxonomy of the features was classified using the USEARCH SINTAX algorithm in RDP training version 16 [23]. The sequencing reads of all samples were rarefied to the sample with the lowest reads to compensate for the difference in the sequencing depth. The α-diversity was determined from the rarefied OTU table based on the richness, Simpson, and Shannon indices. The β-diversity was estimated from the Bray–Curtis distances, which were applied to build distance matrices and reported according to the PCoA. PICRUSt2 was used to predict altered KEGG pathways based on 16S sequencing data [24]. For differential abundance analysis and association analysis, all bacterial taxa with a relative abundance of < 0.01% and present in < 20% of samples were removed.

### Metatranscriptomic analysis

Total microbial RNA was extracted from the colonic contents using the E.Z.N.A.® Soil RNA Midi Kit (Omega Bio-Tek, USA) following the manufacturer’s instructions. The RNA concentration and purity were quantified using a NanoDrop 2000 (Thermo Fisher Scientific, USA). Only samples with an RNA integrity number (RIN) > 7 were used to generate libraries. Total RNA was subjected to ribosomal RNA removal using the Ribo-Zero rRNA Removal Kit (Illumina, San Diego, USA). TruSeq™ RNA Sample Prep Kit (Illumina) was used to construct cDNA libraries. Paired-end sequencing was performed with the barcoded libraries on the Illumina HiSeq 2500 platform at Majorbio Bio-Pharm Technology (Shanghai, China) using the HiSeq 4000 PE Cluster Kit and HiSeq 4000 SBS Kits according to the manufacturer’s instructions.

Raw sequencing reads first went through quality control using Trimmomatic [25], and clean reads were then aligned to the mouse genome and the Silva database with Bowtie2 [26] to exclude host and ribosomal RNA contamination. Quality-filtered metatranscriptomic reads were de novo assembled for each sample with Trinity (v2.2.0) [27]. All contigs were clustered using CD-HIT (v 4.5.8) [28] to obtain unigenes. The abundance of unigenes in each sample was estimated by calculating the transcripts per million (TPM) value based on the number of aligned reads using Salmon (v 0.9.1) [29]. Protein coding sequences were predicted from contigs using MetaGeneMark [30]. For functional annotation, protein sequences were annotated against those of evolutionarily conserved genes in the nonsupervised orthologous groups (eggNOG) using DIAMOND (v.0.8.23) [31] under default settings. Differential enrichment of genes and functions were determined by DESeq2 [32]. Taxonomic classifications were aligned against the NCBI taxonomy database using DIAMOND (v.0.8.23) [31] with default parameters. Then, the blastx results were analyzed using the longReads LCA algorithm of MEGAN [33].

### RNA sequencing and data analysis

Total RNA from the colonic tissue was extracted and purified using TRIzol reagent (Invitrogen). Only those samples with RIN > 7 were used for RNA-seq. TruSeq™ RNA Sample Prep kit (Illumina, USA) was used to construct cDNA libraries. The barcoded libraries were sequenced on the Illumina HiSeq 2500 platform at Majorbio Bio-Pharm Technology (Shanghai, China). Paired-end raw sequencing reads were first quality-controlled by FastQC and mapped to the mouse genome using hisat2 (v 2.1.0) [34]. The low-expression transcripts with an average count < 20 were removed. Analysis of differential gene expression was performed using the DESeq2 package [32]. Differentially expressed genes (DEGs) were defined as genes with a minimum twofold change and a false discovery rate < 0.05. KEGG pathway functional enrichment analysis of DEGs and gene set enrichment analysis (GSEA) of gene ranks were performed using clusterProfiler [35].

### Weighted correlation network analysis

To identify modules of coabundant host genes and bacterial taxa in RNA-seq and 16S rDNA sequencing data, we applied the R package WGCNA [36]. The correlation matrix quantified the interconnectedness between genes or taxa and then assigned them to coabundance modules. The genes or taxa that did not show high enough coexpression metrics with any module were removed. The signed network was derived based on a biweight midcorrelation (bicor) method. A scale-free topology criterion was used to choose soft thresholds of 12 and 5 for gene and taxon correlations, respectively. To identify the modules of genes or taxa that were most relevant in the context of colonic inflammation, correlations between serum tryptophan metabolites and each module were tested with Spearman rank.

### Analysis of the human IBD metatranscriptomic datasets

Publicly available IBD metatranscriptomic datasets were used to determine the differences in microbial genes in tryptophan metabolic pathways. The datasets were obtained from the Sequence Read Archive (BioProject: PRJNA389280), which consists of the human HMP2 metatranscriptomic sequencing reads in the Inflammatory Bowel Disease Multiomics Database [37]. The reads used in the present study were from 46 patients with Crohn’s disease (CD), 21 patients with ulcerative colitis (UC), and 11 healthy controls. The data was processed and analyzed similarly to the mouse metatranscriptomic data as described above. For the IBD cohort, linear mixed effect models were further applied to determine the abundance of diagnosed microbial DEGs as follows: gene ~ (intercept) + diagnosis + antibiotics + chemotherapy + immunosuppressant. Diagnosis was coded as a categorical variable (CD, UC, non-IBD) with non-IBD as the reference state. The three medical treatments were coded as binary covariates with nonuse as the reference state. Gene abundance values were log-transformed and zero-smoothed prior to linear modeling. All *P* values of multiple comparisons in the analysis above were adjusted to FDR. The threshold of significance was set at *P* < 0.05 (*P < 0.05, **P < 0.01, ***P < 0.001, ****P < 0.0001); ns, not significant; *P* < 0.1 was considered a trend.

### Statistics analysis

QIIME v.2.0, GraphPad Prism (v 6.0), and the R program (v 3.6.1) were used for statistical analysis. The difference in bacterial abundance was evaluated using one-way ANOVA and Tukey’s post hoc test for data with a normal distribution or the Kruskal–Wallis test for data with a nonnormal or nonparametric distribution. *P* < 0.05 was considered statistically significant. When applicable, *P* values were corrected for multiple testing with the Benjamini–Hochberg false discovery rate (FDR) with a cutoff value of 0.1.

## Results

### L. reuteri I5007 protects mice from colitis through modulating the intestinal microbiota

We previously showed *L. reuteri* I5007 to be capable of ameliorating colitis and correcting microbiota in mice and sows [14, 38]. To investigate whether the intestinal microbiota is involved in *L. reuteri-*mediated alleviation of colitis, a colitis-recovery-colitis-colonic microbiota transplantation (CMT) model was employed (Additional file 1: Fig. S1A). Briefly, mice were orally administered with or without *L. reuteri* I5007 for 3 weeks, followed by induction of colitis with DSS for another week. Mice were then allowed to fully recover from colitis for 12 days before a second round of 7-day DSS treatment and reciprocal transplantation of the colonic microbiota of DSS-treated mice administered with and without *L. reuteri* I5007. As expected, *L. reuteri* I5007 significantly alleviated the disease activity index (DAI) and colonic pathology in the first round of DSS treatment, while recovery for 12 days completely restored intestinal health in both groups of DSS-treated mice (Additional file 1: Fig. S1B-D). Interestingly, the colonic microbiota of DSS-treated mice was largely restored to normal, while that of *L. reuteri* I5007-supplemented mice remained significantly divergent (*p* < 0.05) on day 40 even after 12 days of recovery (Additional file 1: Fig. S1E). The α-diversity indices of the colonic microbiota as indicated by richness and Shannon Index remained drastically reduced in DSS-treated mice administered with *L. reuteri* on day 40 (Additional file 1: Fig. S1F).

Transplantation of day-28 colonic microbiota from *L. reuteri*-administered mice significantly increased the survival and reversed the weight loss of DSS-treated mice (*p* < 0.05) (Additional file 1: Fig. S2A and B). Similarly, DSS-treated mice receiving *L. reuteri*-supplemented microbiota showed decreased pathology scores and increased colon length (Additional file 1: Fig. S2C-E). Moreover, the colonic microbiota from *L. reuteri*-supplemented mice significantly decreased Th17A cells and increased regulatory T cells in the colonic lamina propria and mesenteric lymph nodes (*p* < 0.05) (Additional file 1: Fig. S2F-I). Collectively, the results suggested that the intestinal microbiota is likely to be involved in *L. reuteri*-mediated protection against intestinal inflammation*.*

### Protective effect of L. reuteri is associated with the production of indole derivatives

**Fig. 1:**
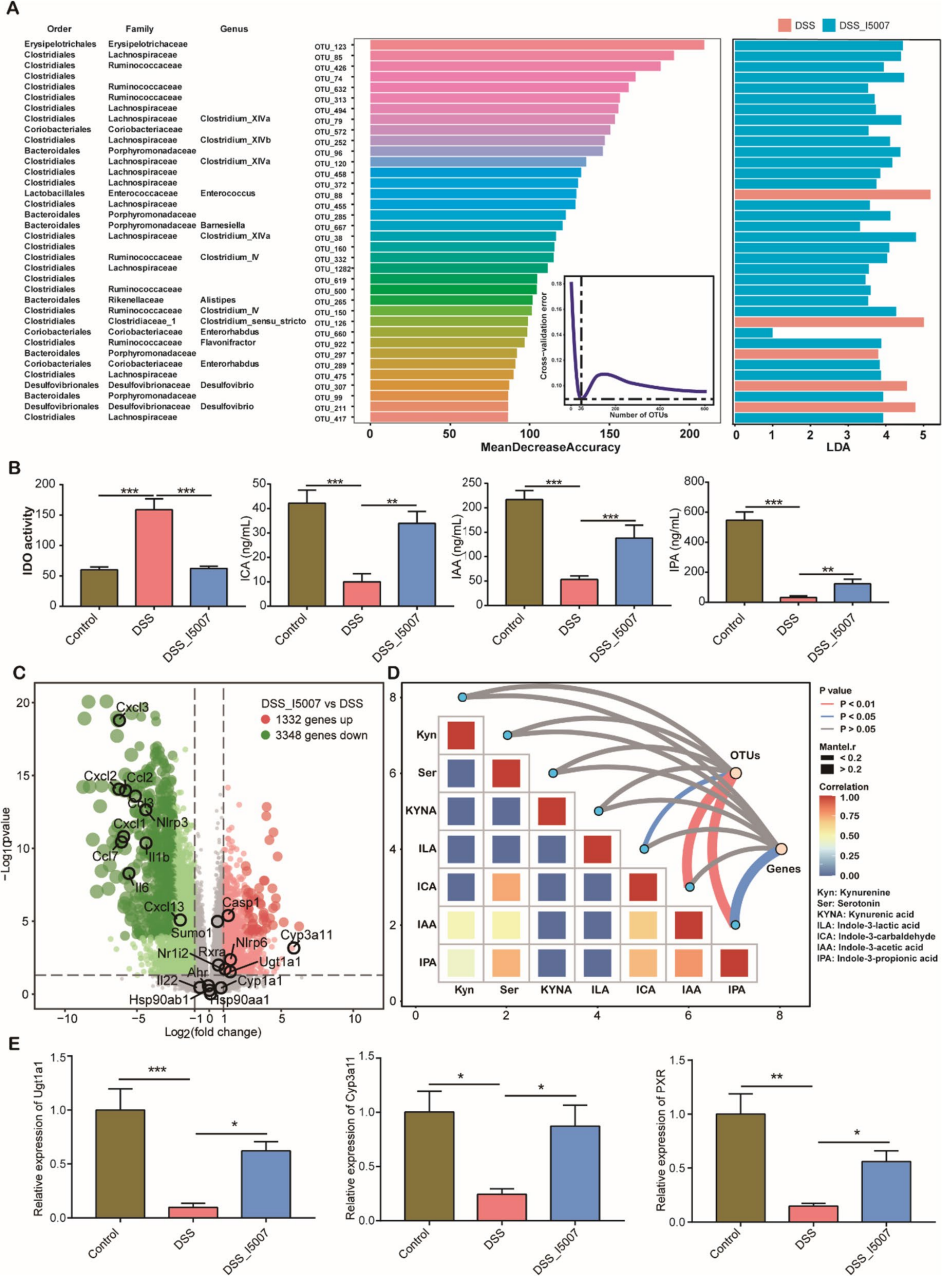
*L. reuteri* alters the intestinal microbiota composition and microbial tryptophan metabolite. **A** Differential enrichment of the mouse colonic microbiota in response to *L. reuteri* supplementation on day 28 (see the Figure S1A legend for the experimental scheme). The taxonomic classifications of the bacteria are shown in the left panel. Blanks are unassigned taxa. The 36 differentially enriched bacterial taxa are ranked by estimating the mean decrease in accuracy based on random forest analysis. The right panel shows the LEfSe analysis of the 36 bacterial taxa. **B** Serum concentrations of metabolites in tryptophan metabolism among three groups of mice (*n* = 10). **C** Volcano plot showing differential gene expression in the colon of DSS-treated mice with or without *L. reuteri* supplementation*.* Each red dot indicates a significantly upregulated gene, while a blue dot represents a significantly downregulated gene, with each gray dot showing a gene with no significant difference. **D** Pairwise comparisons of tryptophan metabolites, with a color gradient denoting Spearman’s correlation coefficient. The correlation between the bacterial/gene expression profiles and each metabolite using partial Mantel tests. The edge width corresponds to Mantel’s R statistic for the corresponding distance correlations, and the edge color denotes statistical significance. **E** Real-time PCR assay showing the expression of *Pxr* and its target genes (*n* = 5). *p < 0.05, **p < 0.01, ***p < 0.001, and ****p < 0.0001

Linear discriminant analysis effect size (LEfSe) analysis was further performed to identify the major colonic bacteria enriched in DSS-treated mice with or without *L. reuteri* I5007 administration. The results showed that 30 bacterial taxa were significantly enriched by *L. reuteri* I5007 (LDA > 3.0, *p* < 0.05) (Fig. 1A). Interestingly, those enriched bacterial taxa were mostly *Clostridium* (*Clostridium XlVa*, *Clostridium XlVb*, and *Clostridium IV*), which are known to be capable of metabolizing tryptophan [39]. Consistently, DSS significantly impaired microbial metabolism of tryptophan as evidenced by reduced levels of indole derivatives such as indole-3-carbaldehyde (ICA), IPA, and IAA in the serum (*p* < 0.01). Conversely, the host metabolism of tryptophan appeared to be enhanced, showing a significant increase in the activity of indoleamine 2,3-dioxygenases (IDO) (*p* < 0.001) (Fig. 1B), key enzymes involved in the kynurenine pathway [40]. On the other hand, administration of *L. reuteri* largely restored the serum concentrations of ICA, IAA, and IPA as well as the IDO activity in DSS-treated mice to healthy levels (Fig. 1B). Interestingly, *L. reuteri* also restored the serum levels of 5-HT and tryptophan, but with no impact on kynurenine or ILA (Additional file 1: Fig. S3A).

RNA sequencing of the colonic tissue further identified 4680 differentially expressed genes in response to *L. reuteri* I5007 in DSS-treated mice (Fig. 1C). KEGG pathway enrichment and gene set enrichment analysis (GSEA) showed that *L. reuteri* administration downregulated inflammation-related pathways such as TNF and NF-κB signaling (Additional file 1: Fig. S3B and C). The correlation analysis of distance dissimilarities further revealed IPA to be in the strongest correlation with both the microbiota and host gene expression, while IAA was significantly correlated only with the microbiota composition (Fig. 1D).

As expected, the expression of *Pxr* (also known as *Nr1i2*), a receptor for IPA [41], and PXR target genes *Ugt1a1* and *Cyp3a11* increased markedly in the colon of DSS-treated mice in response to *L. reuteri* (Fig. 1E). IAA is an AhR ligand that promotes IL-22 production [2, 42]. However, RNA-seq analysis and RT–PCR assays failed to reveal conspicuous differences in the colonic expression of *Ahr* and *Cyp1a1* with and without *L. reuteri* (Fig. 1C, Additional file 1: Fig. S3D, Additional file 2: Table S1). The serum level of IL-22 was unaffected by *L. reuteri* as well (Additional file 1: Fig. S3D). Weighted gene coexpression network analysis showed that there were common host genes and bacterial taxa modules that were significantly correlated with IPA and IAA (Additional file 1: Fig. S3E and F).

### Both IPA and IAA ameliorate DSS-induced colitis

To directly evaluate the protective effect of major microbial tryptophan metabolites on intestinal inflammation and mucosal barrier function, 20 and 40 mg/kg IAA or IPA were separately administered to mice with or without DSS treatment for 7 days (Additional file 1: Fig. S4A). Both IPA and IAA significantly reversed DSS-induced weight loss, increased colon length, reduced colonic pathology, increased goblet cells, and decreased *IL-1β* and *TNF-α* gene expression in the colon, while augmenting the protein expression of E-cadherin and occludin (Additional file 1: Fig. S4B-G), suggesting that both IAA and IPA are capable of alleviating intestinal inflammation and enhancing mucosal barrier function. Similar to *L. reuteri*, IAA failed to enhance the expression of *Ahr* and *Cyp1a1*, while IPA significantly increased the expression of *Pxr* (Additional file 1: Fig. S4H).

**Fig. 2:**
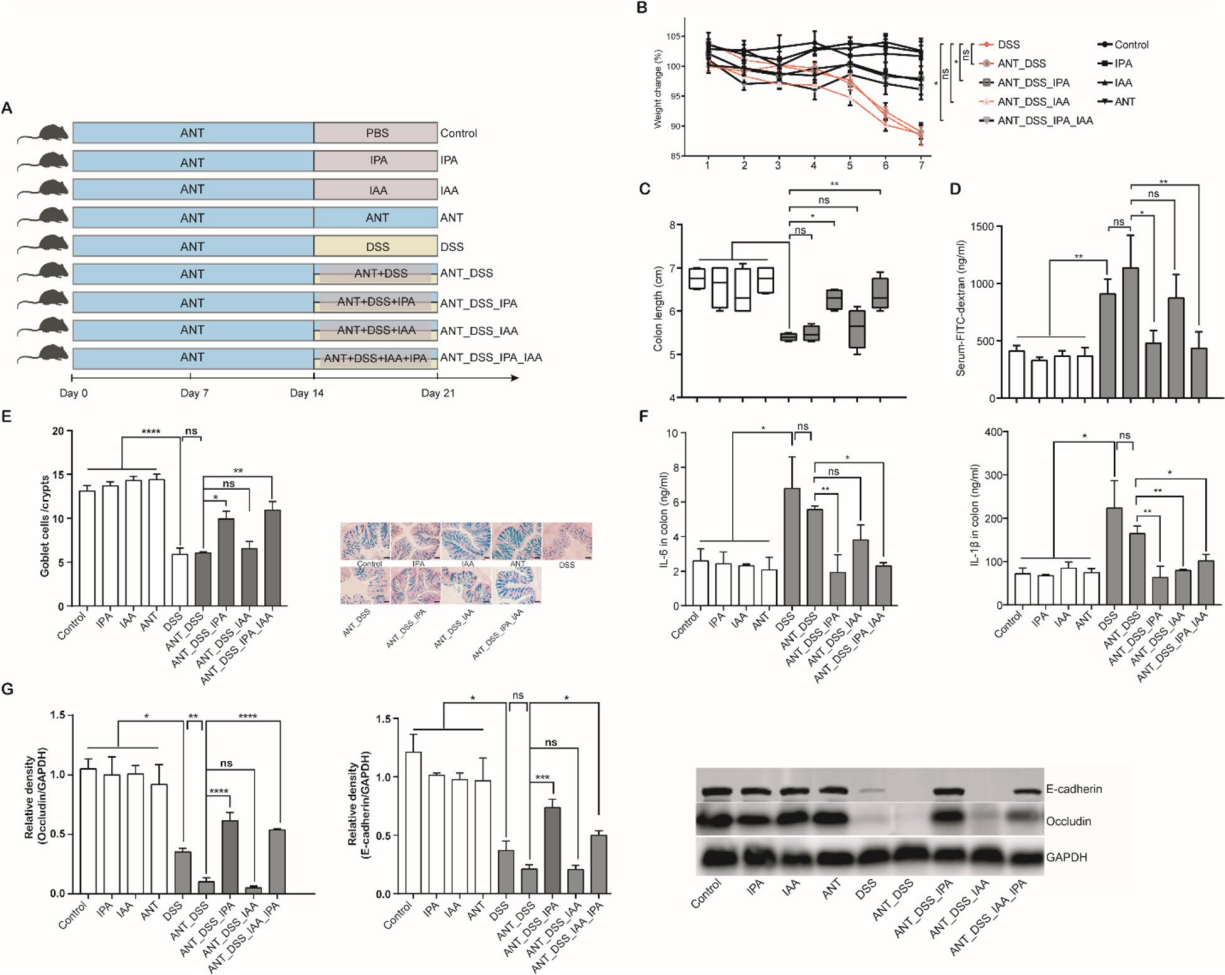
IPA and IAA ameliorate DSS-induced colitis. **A** Experimental scheme of the mouse trial (*n* = 5). Six-week-old C57BL/6 mice were administered with a cocktail of antibiotics for 2 weeks, followed by oral administration of 40 mg/kg IPA and/or IAA daily with or without 3% DSS in drinking water for another 7 days. The severity of colitis was assessed by determining the changes in body weight (**B**) and colon length (**C**). **D** Serum FITC-dextran levels among different groups of mice (*n* = 4). **E** Goblet cell changes in the colon of mice. The left panel shows the goblet cells per crypt of the colon, and the right panel shows representative images of alcian blue staining for goblet cells within the inner mucus layer of colonic sections. **F** Levels of IL-6 and IL-1β in the colonic tissue using ELISA (*n* = 4). **G** The levels of occludin and E-cadherin proteins determined by Western blotting (*n* = 3). *p < 0.05, **p < 0.01, ***p < 0.001, ****p < 0.0001; ns, not significant

Colonic microbiome analysis further revealed that IAA significantly altered both α- and β-diversities of the colonic microbiota of DSS-treated mice, while IPA only had a marginal effect (Additional file 1: Fig. S5A and B), suggesting that the microbiota is differentially regulated by IPA and IAA. To further assess whether IPA- and IAA-mediated protection of DSS-treated mice is dependent upon the intestinal microbiota, mice were treated with antibiotics for 2 weeks to deplete the intestinal microbiota, followed by oral administration of IPA, IAA, or a mixture of IPA and IAA with or without DSS treatment (Fig. 2A). Clearly, IPA or the IPA/IAA mixture was capable of protecting antibiotic-treated mice from DSS-induced colitis, while the protective effect of IAA, as indicated by weight loss, the colon length, intestine barrier, and inflammation was abrogated (Fig. 2B–G), suggestive of a differential involvement of the intestinal microbiota in the protection of mice from intestinal inflammation by IPA and IAA.

### L. reuteri alters the microbial tryptophan metabolism through synthesis of ILA

**Fig. 3:**
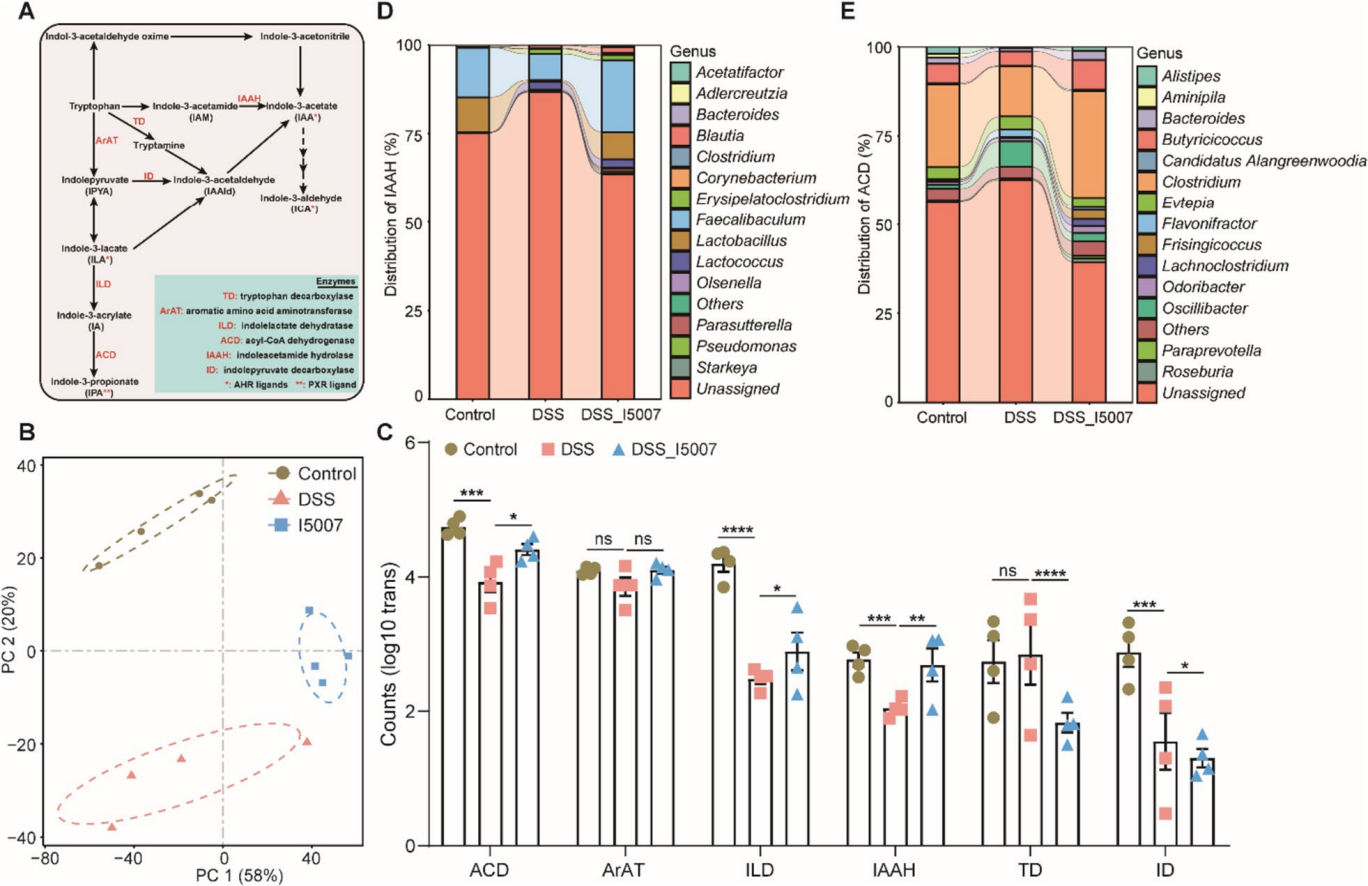
*L. reuteri* alters the expression of major enzymes involved in microbial metabolism of tryptophan. Six-week-old C57BL/6 mice were orally gavaged with 200 µl of PBS with or without *L. reuteri* I5007 (10^9^ CFU/mL) daily for 3 weeks, followed by a week of 3% DSS administration in drinking water to induce colitis (See Figure S1A for experimental scheme). Metatranscriptomics was performed with the colonic microbiota collected on day 28. **A** Major metabolic pathways and enzymes involved in microbial tryptophan metabolism. **B** PCoA plot of the Bray–Curtis distance showing the differences in the colonic microbiota function. **C** The differences in mRNA expression levels of major enzymes involved in microbial tryptophan metabolism among three groups of mice. Statistics was performed using DESeq2. *p < 0.05, **p < 0.01, ***p < 0.001, ****p < 0.0001; ns, not significant

Although *L. reuteri* significantly increased the serum concentrations of IPA and IAA in DSS-treated mice (Fig. 1B), ILA, but not ICA, IPA, or IAA, was found to be synthesized by *L. reuteri* and several other *Lactobacillus* species (Additional file 1: Fig. S6A), consistent with earlier observations with *Lactobacillus* and *Bifidobacterium* [13]. Tryptophan supplementation of the *L. reuteri* culture also significantly increased ILA synthesis (Additional file 1: Fig. S6B). Microbial tryptophan metabolism involves multiple enzymatic reactions, and ILA is a critical intermediate (Fig. 3A). Metatranscriptomic analysis of the colonic microbiota from DSS-treated mice supplemented with or without *L. reuteri* I5007 revealed that, while DSS suppressed the expression of most bacterial genes involved in tryptophan metabolism, *L. reuteri* significantly increased the mRNA expression of acyl-CoA dehydrogenase (ACD), indolelactate dehydrogenase (ILD), and indoleacetamide hydrolase (IAAH), with reduced expression of indolepyruvate decarboxylase (ID) (Fig. 3B, C), suggesting that *L. reuteri* prefers the synthesis of ILA, IPA, and IAA. Additionally, the relative abundance of *Clostridium*, which encodes both ACD and IAAH, was increased upon *L. reuteri* administration (Fig. 3D, E).

### ILA ameliorates DSS-induced colitis and alters the intestinal microbiota composition

To directly assess the efficacy of ILA in alleviating intestinal inflammation, DSS-treated mice were orally administered daily with or without ILA for a week. ILA significantly reversed DSS-induced weight loss (Additional file 1: Fig. S7A) and colon length shortening (Additional file 1: Fig. S7B). Consistently, ILA significantly suppressed DSS-induced production of TNF-α and IL-1β in the serum (Additional file 1: Fig. S7C). Colonic histological scores were also significantly reduced in ILA-treated mice (Additional file 1: Fig. S7D), as indicated by lessened ulceration, edema, crypt damage, and immune cell infiltration of the intestinal epithelial layer (Additional file 1: Fig. S7E). ILA also partially restored DSS-induced reduction of richness and Shannon index (Additional file 1: Fig. S7F) of the colonic microbiota. Notably, ILA further caused a clear segregation of the colonic microbiota in DSS-treated mice (Additional file 1: Fig. S7G). At the genus level, ILA restored relative abundance of *Clostridium_*XlVa (from 6.3 to 35.8%) and *Lactobacillus* (from 0.45 to 3.8%) in DSS-treated mice (Additional file 1: Fig. S7H).

To further examine whether the protective effect of ILA is dependent upon the intestinal microbiota, mice were treated with antibiotics for 2 weeks to deplete the intestinal microbiota, followed by DSS treatment with or without ILA supplementation (Additional file 1: Fig. S8A). The results showed that the protective effects of ILA on weight loss and colon shortening were significantly diminished in the microbiota-depleted mice, relative to the conventional mice (Additional file 1: Fig. S8B-E). ILA is known to exert its protective effect by activating AhR [11, 13]. To examine whether pharmacologic inhibition of AhR abrogates ILA-mediated amelioration of intestinal inflammation, mice were administrated with CH223191, an AhR inhibitor, together with ILA during DSS treatment (Additional file 1: Fig. S8F). To our surprise, ILA conferred significant protection even in the presence of CH223191, as indicated by weight loss, the colon length, and histopathology (Additional file 1: Fig. S8G-J). Therefore, we conclude that the microbiota modulation may be another pathway to AhR activation for IAA to exert the protective effect on intestinal inflammation.

*ILA, IPA, and IAA alleviate intestinal inflammation in IL-10* ^−*/*−^ *mice.*

**Fig. 4:**
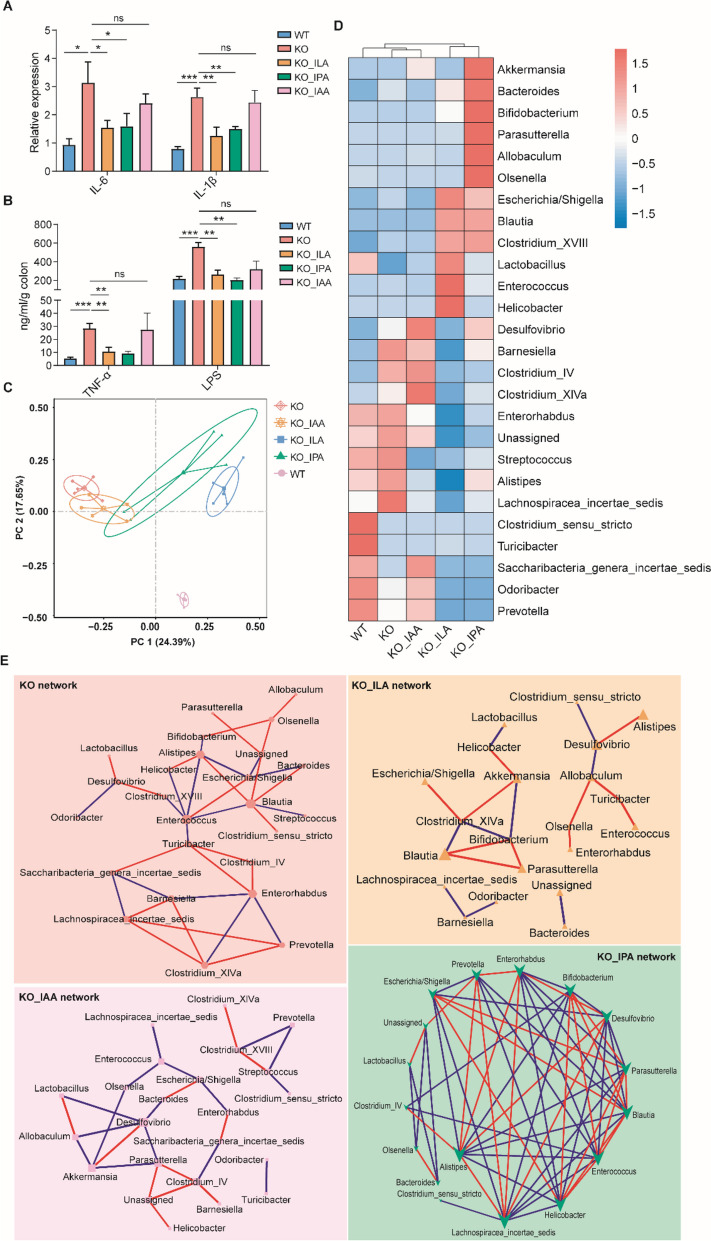
Indole derivatives alleviate intestinal inflammation in IL-10^−/−^ mice. Six-week-old IL-10^−/−^ (KO) mice were orally gavaged daily with or without ILA (20 mg/kg), IPA (40 mg/kg), or IAA (40 mg/kg) for 4 weeks (*n* = 4). Congenic wild-type (WT) mice were also gavaged daily for 4 weeks as negative controls. **A** Colonic mRNA expression levels of proinflammatory cytokines (IL-6 and IL-1β) (*n* = 4) by RT-qPCR. **B** The levels of TNF-α and LPS in the colon (*n* = 4) using ELISA. **C** PCoA plot of the Bray–Curtis distance of the colonic microbiota among different groups of mice. **D** Heatmap showing the relative abundance of different bacterial genera. **E** Co-occurrence network analysis of bacterial genera among different groups of mice. Edges representing significant SparCC correlations indicate |*r*|> 0.6 and *p* < 0.05. Each light blue line represents a significant negative correlation, while each light red line represents a significant positive correlation. The size of the points represents the degree of the node. The thickness of the line is proportional to the degree of correlation. *p < 0.05, **p < 0.01, ***p < 0.001, ****p < 0.0001; ns, not significant

IL-10^−/−^ mice are known to spontaneously develop colitis [43]. To confirm whether ILA, IPA, and IAA are capable of protecting mice from intestinal inflammation in a different colitis model, ILA, IPA, or IAA was orally administered to IL-10^−/−^ mice for 4 weeks. Both ILA and IPA significantly suppressed the colonic expression of *IL-1β* and *IL-6* mRNA, while IAA has a lesser effect (Fig. 4A). ILA and IPA were also capable of restoring TNF-α and LPS to healthy levels in the colon, with a marginal effect observed with IAA (Fig. 4B). Overall, these results suggested that ILA and IPA are more efficient than IAA in protecting mice from intestinal inflammation in both DSS and IL-10^−/−^ murine models of colitis. Additionally, ILA and IPA altered the colonic microbiota of IL-10^−/−^ mice more drastically than IAA (Fig. 4C). Importantly, ILA supplementation markedly elevated relative abundance of *Lactobacillus* (from 6.45 to 39.58%), while IPA enriched *Bifidobacterium* (from 0.01 to 0.81%) and *Akkermansia* (from 0.21 to 10.37%) (Fig. 4D). ILA and IPA also increased relative abundance of *Blautia* (from 0.06 to 1.25% and 1.22%, respectively). The co-occurrence networks revealed *Blautia* and *Bifidobacterium* to be key bacterial taxa in the microbial networks in response to ILA and IPA (Fig. 4E). Notably, IPA had the most complex network of the microbial features (Fig. 4E). Taken together, these results suggested that the intestinal microbiota is likely involved in ILA- and IPA-mediated alleviation of colitis.

### *ILA increases IPA and IAA production through microbial cross-feeding both *in vivo* and *in vitro

**Fig. 5:**
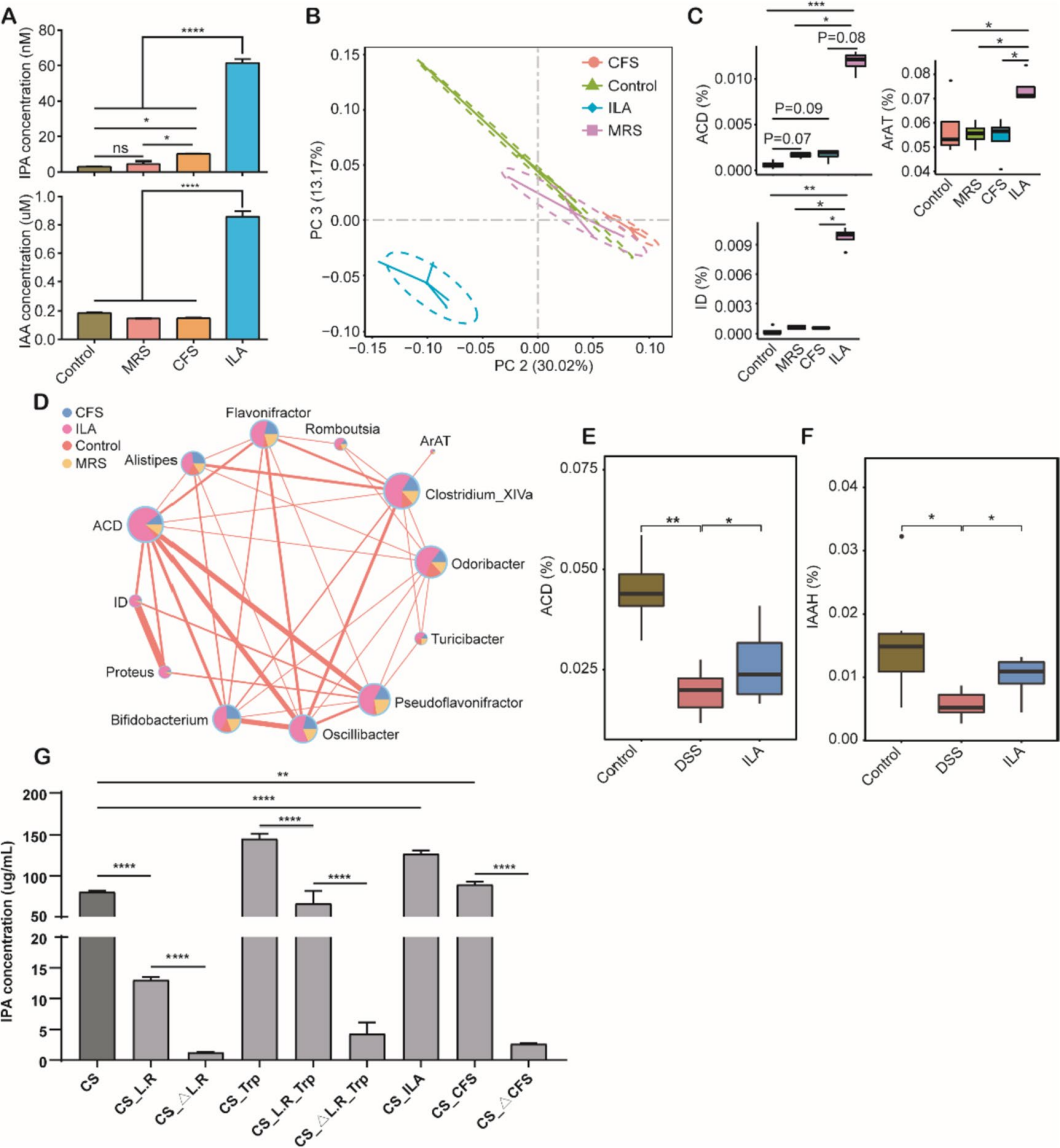
ILA promotes the microbial production of IPA and IAA in vitro. The colonic microbiota was cultured under anaerobic conditions with or without an addition of de Man Rogosa Sharpe (MRS) medium, cell-free supernatant (CFS) of *L. reuteri* I5007, or ILA for 12 h at 37 °C. **A** The concentrations of IPA and IAA in the anaerobic culture supernatant of the colonic microbiota (*n* = 4). **B** PCoA plot of the Bray–Curtis distance among different treatment groups (*n* = 4). **C** Relative abundance of three major enzymes involved in microbial tryptophan metabolism as predicted by PICRUSt2. **D** Network analysis of differentially enriched bacterial genera and enzymes. Edges representing significant Spearman’s correlations indicate |*r*|> 0.7 and *p* < 0.05. The thickness of each line is proportional to the magnitude of the correlation. The pie chart shows the relative abundance (%) of bacterial genera or enzymes among different groups. ArAT, aromatic amino acid aminotransferase; ID, indolepyruvate decarboxylases. Relative abundances of acyl-CoA dehydrogenase (ACD) (**E**) and indoleacetamide hydrolase (IAAH) (**F**) predicted by PICRUSt2 among DSS-treated mice administered with or without ILA (see the Figure S7 legend for experimental details). **G** The *C. sporogenes* was cultured with or without an addition of Tryptophan, *L. reuteri* I5007, ArAT-deficient mutant *L. reuteri* I5007(△L.R), CFS of *L. reuteri* I5007 or △L.R, ILA for 24 h at 37 °C (*n* = 4). *p < 0.05, **p < 0.01, ***p < 0.001, ****p < 0.0001; ns, not significant

Because only ILA was the only tryptophan metabolite synthesized by *L. reuteri* (Additional file 1: Fig. S6), enhanced synthesis of IPA and IAA in L. *reuteri-* supplemented DSS mice was likely a result of utilization of ILA as a substrate by other intestinal bacteria. To directly confirm whether ILA increases IPA and IAA production through microbial cross-feeding, we incubated ILA with the colonic microbiota of DSS-treated mice and found ILA significantly increased IPA and IAA levels (Fig. 5A). The microbiome analysis indicated that ILA markedly altered the composition of the microbiota (Fig. 5B). Functional metagenomic prediction by PICRUSt2 showed that the expression of ACD, ID, and ArAT increased in response to ILA (Fig. 5C). The coabundance network revealed that ACD had the highest degree of the interactions showing a strong positive correlation with several differentially enriched bacterial genera (Fig. 5D). Furthermore, oral administration of ILA to DSS-treated mice also significantly increased the ACD and IAAH expression in the colonic microbiota as predicted by PICRUSt2 (Fig. 5E, F). To further verify that ILA promotes the production of other indole derivatives, we co-cultured tryptophan, *L. reuteri* I5007, CFS and ILA with *C. sporogenes*, which is a specific IPA-producing bacterium. As expected, ILA and CFS of *L. reuteri* markedly increased the production of IPA, but not IAA (Fig. 5G). Collectively, these data suggested that ILA may be utilized by bacteria as a substrate to produce IPA and IAA.

**Fig. 6:**
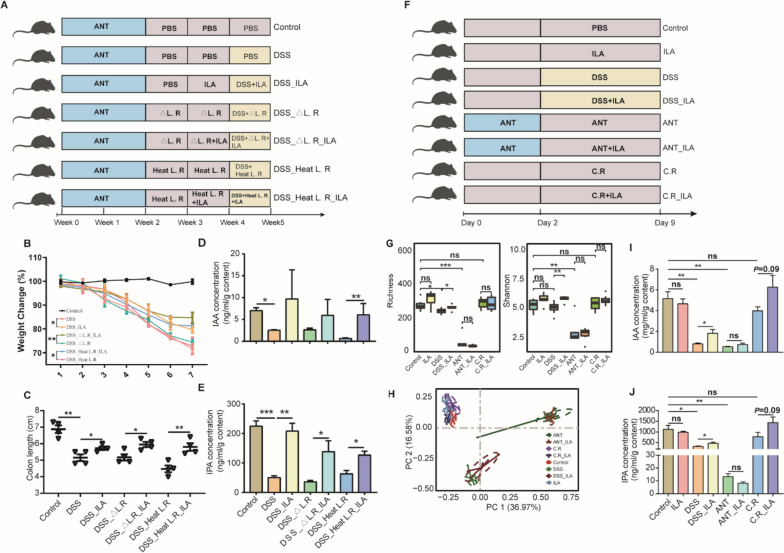
ILA promotes the microbial synthesis of IPA and IAA. **A** Experimental scheme to examine the role of cross-feeding (*n* = 4). The intestinal microbiota was depleted by 2 weeks of antibiotic administration in the drinking water, followed by oral gavage of ArAT-deficient mutant (△L.R) or heat-killed *L. reuteri* for another 2 weeks with or without oral administration of 40 mg/kg ILA in the second week. Colitis was induced by adding 3% DSS in drinking water in the fifth week. Colitis severity was assessed by determining the changes in body weight (**B**) and colon length (**C**). The concentrations of IAA (**D**) and IPA (**E**) were measured in the colon contents. **F** Experimental scheme to evaluate the role of the intestinal microbiota on ILA-mediated synthesis of IPA and IAA. Mice were administered with or without a cocktail of antibiotics for 2 days, followed by induction of dysbiosis for a week (*n* = 7 or 8). Three different dysbiosis models were employed including 3% DSS in drinking water, antibiotic cocktail in drinking water, and oral daily challenged with 10^8^ CFU/ml *C. rodentium*. **G** Richness and Shannon Index of the colonic microbiota among different groups of mice on day 9. **H** PCoA plot of the Bray–Curtis distance. Comparisons of the concentrations of IAA (**I**) and IPA (**J**) in the colon contents among groups are shown (*n* = 5). *p < 0.05, **p < 0.01, ***p < 0.001, ****p < 0.0001; ns, not significant

To further examine whether ILA promotes IPA and IAA production through microbial cross-feeding in vivo, we treated mice with antibiotics to deplete the intestinal microbiota, followed by supplementation of heat-killed or an *ArAT*-deficient *L. reuteri* mutant strain (ΔArAT) with or without oral administration of ILA (Fig. 6A). Both heat-killed and ΔArAT *L. reuteri* failed to protect mice against DSS-induced colitis (Fig. 6B, C). Notably, the heat-killed and ΔArAT *L. reuteri* cannot promote the production of IPA and IAA, while administration of ILA to mice receiving heat-killed or ΔArAT mutant strain led to increased syntheses of both metabolites in the colonic content (Fig. 6D, E). These results confirmed that ILA is the major tryptophan metabolite produced by *L. reuteri* to be responsible for microbial synthesis of IPA and IAA in the GI tract.

To further verify whether ILA enhances IPA and IAA production dependent upon the intestinal microbiota, we either disrupted the microbiota with antibiotics or induced dybiosis with *Citrobacter rodentium* or DSS with or without oral administration of ILA (Fig. 6F). The microbiome analysis showed that ILA had no impact on microbial α- and β-diversity of healthy, *C. rodentium*-challenged, or antibiotic-treated mice (Fig. 6G, H). Notably, ILA remained largely effective in both dysbiotic mouse models. ILA significantly enhanced IPA and IAA in DSS-treated mice (*p* < 0.05), with a strong tendency (*p* = 0.09) to increase IPA and IAA in *C. rodentium*-challenged mice; however, ILA failed to promote the synthesis of either IPA or IAA in the antibiotic-treated mice (Fig. 6G, H), suggesting clearly that the intestinal microbiota is required for ILA-induced IPA and IAA synthesis. Collectively, these results indicated that ILA enhances IPA and IAA production through microbial cross-feeding.

### Alterations in microbial tryptophan metabolism in IBD patients

Similar to our observations in DSS-induced colitis (Fig. 1B), host tryptophan metabolism is enhanced in IBD patients [44]. However, it is unclear whether microbial metabolism of tryptophan is suppressed in IBD patients as in DSS-treated mice. To examine the expression levels of the major bacterial genes responsible for tryptophan metabolism in IBD patients, we retrieved the metatranscriptomic profiling datasets from the HMP2 IBDMDB cohort, which includes the stool samples from both ulcerative colitis (UC, *n* = 21) and Crohn’s disease (CD, *n* = 46) patients as well as healthy controls (*n* = 11) [45]. PCoA plot showed that the microbiota profile responsible for tryptophan metabolism are different from healthy controls, with no obvious difference between UC and CD patients (Additional file 1: Fig. S9A). Among the microbial metabolic genes, only ACD showed a significant reduction in individuals with UC (Additional file 1: Fig. S9B). ACD was mainly produced by *Faecalibacterium* (21.9%), *Roseburia* (20.7%), and *Prevotella* (7.5%) in the stool samples, and their abundances were substantially reduced in IBD patients (Figure S9C and 9E). At the species level, three most abundant bacteria including *Roseburia* sp*.* CAG:18_43_25 (13.2%), *Faecalibacterium prausnitzii* (9.6%), and *Prevotella copri* (8.8%), were markedly reduced in IBD patients (Additional file 1: Fig. S9D and F). Suppression of ACD and ACD-producing bacteria is consistent with an earlier observation on IPA reduction in IBD-induced dysbiosis [46]. Collectively, these results strongly suggested that tryptophan metabolism by the intestinal microbiota is very likely to be reduced in IBD patients.

## Discussion

In this study, we characterize a molecular mechanism whereby metabolites contribute to the modulation of a symbiotic intestinal microenvironment. Specifically, microbial tryptophan metabolites ILA play a critical role in mediating inter-microbial communication and cooperatively control intestinal homeostasis with other microbiota-derived indoles. Our results demonstrate that ILA increased microbial tryptophan metabolism and promotes the synthesis of IPA and IAA in vivo and in vitro. We further identified several bacteria such as *Clostridium* to metabolize ILA to IPA and IAA by inducing the expression of several core tryptophan-metabolizing enzymes. Furthermore, ILA-mediated microbial cross-feeing is microbiota-dependent and specifically enhanced indoles levels under conditions of dybiosis inducing by *Citrobacter rodentium* or DSS, but not of microbiota disruption with antibiotics.

Consistent with an earlier study [47], we found that *L. reuteri* dramatically enriches *Clostridium XIVa* and *Clostridium IV*, which are responsible for the proliferation of CD4^+^CD25^+^Foxp3^+^ Treg cells in the colonic mucosa [48]. Transplantation of the colonic microbiota of *L. reuteri-*administered mice recapitulated the protective effect of *L. reuteri* against colitis in DSS-treated mice. The metabolomics analysis revealed that *L. reuteri* reverses the impaired microbial tryptophan metabolism in colitis mice by enhancing the expression of metabolizing enzymes leading to increased IPA and IAA syntheses. In agreement with a previous report [41], our data indicated that IPA significantly enhances epithelial barrier integrity and decreases inflammation in two different murine models of intestinal inflammation including DSS-induced colitis and IL-10^−/−^ spontaneous colitis. Our results showed that IAA may alleviate colitis by modulating the intestinal microbiota and does not rely exclusively on the activation of AhR in DSS mice. The previous research also reported that the chemotherapy efficacy of IAA is licensed by immune cell-derived myeloperoxidase, but not by AhR signaling [4]. Notably, *L. reuteri* elevated the transcript levels of PXR and related genes without activating AhR and its target genes. These data suggested that the protective properties of IAA against colitis may be dependent upon the intestinal microbiota, but those of IPA rely on PXR activation.

The underlying molecular mechanism of ILA in maintaining intestinal homeostasis is largely unknown [49]. Our data indicated that ILA reduces the intestinal inflammation and pathologies in DSS-induced colitis. ILA is known to inhibit inflammation by decreasing proinflammatory cytokine levels via activation of AhR [13, 49]. However, we showed that the protective effect of ILA is largely unaffected by an AhR antagonist in DSS-treated mice. Our microbiota analysis further revealed that ILA markedly altered the intestinal microbiota, with a significant enrichment of *Clostridium XIVa*, a major tryptophan-metabolizing bacterium [39]. Additionally, antibiotic-treated colitis mice failed to respond positively to ILA administration. Furthermore, ILA alleviated the intestinal inflammation and altered the microbiota composition in IL-10^−/−^ mice. These results suggested that ILA protects against colitis and intestinal inflammation by modulating the intestinal microbiota, consistent with an earlier report that *L. reuteri*-mediated differentiation of the intestinal intraepithelial lymphocytes is ILA-dependent and relies on the presence of the intestinal microbiota [11].

Bacterial cross-feeding is the utilization of metabolites from one bacterium by another, which is ubiquitous in natural communities and increases the diversity of the intestinal microbiota [50]. We showed in the study for the first time that *Lactobacillus* with the ability of modulating intestinal immunity and gut microbiota through the synthesis of ILA, which is a “core” intermediate metabolite in the bacterial tryptophan metabolism pathways [51]. First, ILA, but not IAA or IPA, was detected in the supernatant of *L. reuteri* and other *Lactobacillus* species. Second, ILA induced IPA and IAA syntheses in both anaerobic culture of the colonic microbiota and the colonic microbiota of DSS-treated mice. Thirdly, ACD and IAAH, two core bacterial enzymes involved in the synthesis of IPA and IAA, and the bacteria known to encode these enzymes (e.g., *Roseburia*, *Faecalibacterium*, and *Clostridium*) [5], were enriched in response to administration of ILA or ILA-producing *L. reuteri.* Fourthly, a mutant strain of *L. reuteri* deficient in ArAT, a major bacterial enzyme responsible for the conversion of tryptophan to ILA [11], lost its ability to produce IPA and IAA or protect mice against DSS-induced colitis. These independent lines of evidence collectively suggested that ILA is very likely to be metabolized by bacteria through cross-feeding to produce IPA and IAA.

In accordance with our results, ILA-producing *Bifidobacterium* has been shown to be positively associated with serum IPA levels especially among lactase nonpersistent individuals [39]. Another study using gnotobiotic mice showed increased concentrations of both IAA and IPA after inoculation with GUT-108, a consortium of 17 live bacterial strains [39], indicating that the production of IAA and IPA could be accomplished cooperatively by intestinal bacteria. A strong positive association between fiber intake and circulating IPA levels was also reported, although IPA is not derived from fiber fermentation [39, 52]. It is plausible that high fiber intake enriches certain tryptophan-metabolizing bacteria such as *Clostridium* to produce IPA through cross-feeding among bacteria. Taken together, ILA is likely to be utilized by intestinal bacteria to promote IPA and IAA production.

The efficacy of cancer chemotherapy or the response to probiotic interventions has been reported to be strongly influenced by individuals intestinal microbiota [4, 53]. Consistently, the regulation of the intestinal microbiome by ILA was microbiota-dependent and specifically enhanced indoles levels under conditions of dybiosis with *Citrobacter rodentium* or DSS inducing, but not of microbiota disruption with antibiotics. This may be related to the reduction of some specific tryptophan-metabolizing bacteria. Using metatranscriptome approach, we confirmed dysbiosis of tryptophan-metabolizing bacteria and the expression of acyl-CoA dehydrogenase, which is required for producing IPA [7], is drastically diminished in IBD patients.

Therefore, our study suggests that microbiota composition and function may be regulated by probiotic approaches. The metabolite treatment mediating downstream microbiota-derived metabolites may more enable to control the host–microbiota interactions. Utilizing the endogenous factors correcting the microbiota from disease to a healthy state may help overcome the strong inter-individual variability, which currently limits the effectiveness of drug treatments or probiotic interventions. The microbiota-derived metabolites or targeted “postbiotic” may provide novel prevention or treatment approaches of dysbiosis-driven diseases.

### Supplementary Information

**Additional file 1: Figure S1.** *L.* *reuteri* protects mice from DSS-induced colitis. (A) Experimental scheme of a colitis-recovery-colitis-colonic microbiota transplantation (CMT) model (*n* = 18-20). Six-week-old C57BL/6 mice were orally gavaged with 200 µl of PBS with or without *L. reuteri* I5007 (109 CFU/mL) daily for three weeks, followed by a week of 3% DSS administration in drinking water to induce colitis and then a 12-day self-recovery period. From day 40-47, a second round of colitis was induced with 2% DSS in drinking water for a week, followed by reciprocal CMT. The mice in the DSS group were gavaged with day-28 colonic bacteria from the DSS_I5007 group, while the mice in the DSS_I5007 group were administered with day-28 colonic microbiota from the DSS group. (B) Dynamic changes in the disease activity index (DAI) score from day 21 to 40. (C) Colonic histological scores of mice (*n* = 6). (D) Representative images of hematoxylin and eosin staining of the colonic sections. (E) PCoA analysis of the Bray–Curtis distances of the colonic microbiota among three groups of mice on day 40. (F) The α-diversity (Richness and Shannon indices) of the colonic microbiota (*n*=8) on day 40. The median value of each group is shown. ****p<0.0001, ***p<0.001, **p<0.01, *p<0.05; ns, not significant. **Figure S2.** The colonic microbiota of *L. reuteri*-supplemented mice protects mice from DSS-induced colitis. Trial design saw the Supplementary Figure 1A legend for experimental details. The survival (A) and body weight changes (B) of mice (*n* = 5) were recorded between day 47-51. Statistical analysis was performed with weight changes using one-way ANOVA and Tukey’s post-hoc test. **p < 0.01 indicates significant differences between DSS_FI5007 and I5007_FDSS groups on days 47 and 48. The histological score (C) and representative images (D) of hematoxylin and eosin-stained colonic sections of different groups of mice on day 51 (*n* = 4). (E) The colon lengths of different groups of mice on day 51 (*n* = 4). Total Th17A cell numbers (F) and Treg cell numbers (G) in the common lymphoid progenitor (CLP) cells as well as Th17A cell numbers (H) and Treg cell numbers (I) in mesenteric lymph nodes (MLNs) were determined by flow cytometry (*n* = 4). *p < 0.05, **p < 0.01, ***p < 0.001, ****p < 0.0001; ns, not significant. **Figure S3.** *L. reuteri* affects the levels of tryptophan metabolites and the expression of AhR-related genes. Trial design saw the Supplementary Figure 1A legend for experimental details. (A) Serum concentrations of tryptophan metabolites (kynurenine, tryptophan, 5-HT, and ILA) (*n* = 10) on day 28 at the end of the trial. (B) Scatter plot of Kyoto Encyclopedia of Genes and Genomes (KEGG) pathway enrichment results of differentially expressed genes. The rich factor indicates the number of differentially expressed genes located in the KEGG pathway. The color represents the significance of the difference. (C) Gene set enrichment analysis (GSEA) results show negative enrichment of inflammation-related gene sets, which were changed after *L. reuteri* treatment compared with the DSS group. (D) Changes in AhR and its target genes (*Cyp1a1* and *IL-22*) in the colon of mice (*n* = 6). (E) Principal component analysis (PCA) plot of functional profiles with gene modules. (F) Correlation network analysis between differentially expressed genes and bacteria based on Spearman’s correlation. Red connections indicate a positive correlation (*r* > 0.4, FDR < 0.05) and blue connections represent negative correlations (*r* < -0.4, FDR < 0.05), while gray connections indicate no correlation (FDR > 0.05). *p < 0.05, **p < 0.01, ***p < 0.001, ****p < 0.0001; ns, not significant. **Figure S4.** IPA and IAA ameliorate DSS-induced colitis in mice. (A) Trial design (*n* = 5). Colitis was induced by providing 3% DSS in the drinking water of mice for 7 days with or without oral administration of 20 or 40 mg/kg IPA or IAA. (B) Body weight changes during the trial. (C) The colon length of mice among different groups of mice on day 7. (D) Histological score (upper panel) and representative images of hematoxylin and eosin staining (lower panel) of the colonic sections on day 7. Scale bars represent 50 µm (*n* = 5). (E) Goblet cell changes in the colon of mice (*n* = 3). The upper panel shows the goblet cells per crypt of the colon, and the lower panel shows representative images of alcian blue staining for goblet cells in the inner mucus layer of colonic sections. (F) Levels of IL-1β and TNF-α mRNAs in the colon (*n* = 5) assessed using RT–qPCR. (G) Expression of the occludin and E-cadherin proteins (*n* = 3) determined by Western blotting. (H) Colonic expression of the *AhR*, *Cyp1a1* and *Pxr* mRNAs (*n* = 3) by RT-qPCR. *p < 0.05, **p < 0.01, ***p < 0.001, ****p < 0.0001; ns, not significant. **Figure S5.** IPA and IAA induce a shift in the colonic microbiota composition of mice. Colitis was induced by providing 3% DSS in the drinking water of mice for 7 days with or without oral administration of 20 or 40 mg/kg IPA or IAA. (A) Boxplots of α-diversity (richness, Simpson, and Shannon indices) of the colonic microbiota on day 7 (*n* = 4). The medians of the data are shown. The statistical analysis was performed using the Wilcoxon rank-sum test. *p < 0.05; ns, not significant. (B) PCoA plot of the Bray–Curtis distance to assess of β-diversity of the colonic microbiota on day 7 (*n* = 4) among different groups of mice. **Figure S6.** Measurement of indole derivatives in the cell-free supernatant. (A) Different *Lactobacillus* species were cultured under anaerobic conditions with an addition of 10% de Man Rogosa Sharpe (MRS) medium for 20 h at 37°C, followed by measurement of four different indole derivatives in the supernatant of each anaerobic culture. (B) *L. reuteri* I5007 was incubated with different concentrations of tryptophan under anaerobic conditions for 20 h at 37°C, followed by measurement of ILA in the supernatant. **Figure S7.** ILA alleviates DSS-induced colitis and alters the intestinal microbiota composition. Six-week-old C57BL/6 mice were orally gavaged with 200 µl of PBS with or without 20 mg/kg ILA daily for one week. Colitis was induced by adding 3% DSS in drinking water. (A) Body weight changes among three groups of mice (*n* = 6). (B) The colon length of mice among three groups of mice (*n* = 6). (C) Levels of TNF-α and IL-1β in the serum (*n* = 3). (D) Histological scores of the colon (*n* = 6). (E) Representative images of hematoxylin and eosin staining of the colonic sections. (F) Richness and Shannon index of the colonic microbiota among three groups of mice. (G) PCoA plot of the Bray–Curtis distance among three groups of mice. (H) Relative abundance of top 15 genera. *p<0.05, **p<0.01, ***p<0.001, and ****p<0.0001. **Figure S8.** ILA alleviates alters the colonic microbiota composition of DSS-treated mice. (A) Trial design. The intestinal microbiota of 6-week-old C57BL/6 mice (*n* = 5) was depleted with a cocktail of antibiotics in drinking water for two weeks, followed by 3% DSS in drinking water with or without oral gavage of 20 mg/kg ILA daily for another week. (B) Weight changes between day 14-21. (C) The colon length of mice among different groups. Histological score (D) and representative images of hematoxylin and eosin staining of the colonic sections (E) were shown. (F) Trial design. Mice (*n* = 5) were orally administered with or without 20 mg/kg ILA for a week, followed by induction of colitis with 3% DSS in drinking water for another week with or without oral supplementation of 20 mg/kg ILA or CH223191. (G) Body weight changes between day 7-14 among different groups of mice. (H) The colon length of mice on day 14 in different groups. Histological score (I) and representative images of hematoxylin and eosin staining of the colonic sections (J) were shown. *p < 0.05, **p < 0.01, and ****p < 0.0001, ns, not significant. **Figure S9.** Bacterial and bacterial enzymes involved in microbial metabolism of tryptophan are altered in IBD patients. Human HMP2 metatranscriptomic sequencing reads of 46 Crohn’s disease (CD) patients, 21 ulcerative colitis (UC) patients, and 11 healthy controls (nonIBD) were retrieved from the Inflammatory Bowel Disease Multiomics Database. (A) PCoA plot of the Bray–Curtis distance along CD, UC, and healthy patients. (B) Normalized abundances of acyl-CoA dehydrogenase (ACD) among CD, UC, and healthy patients. Statistical significance was determined from linear mixed effects models. *p < 0.05. (C) Distribution of ACD in the top 10 genera (C) and species (D). The changes of top 10 ACD-expressing bacterial genera (E) and species (F) among CD, UC, and healthy patients.  

**Additional file 2: Table S2**. Primer sequences used in the present study.

## Data Availability

16S rDNA and metatranscriptomic sequencing data in the present study have been deposited in the NCBI Sequence Read Archive database under accession numbers PRJNA782376 and PRJNA782681.

## Abbreviations

AhR: Aryl hydrocarbon receptor
ACD: Acyl-CoA dehydrogenase
ArAT: Aromatic amino acid aminotransferase
CD: Crohn’s disease
CFS: Cell-free supernatant
CLP: Common lymphoid progenitor
CMT: Colonic microbiota transplantation
DAI: Disease activity index
GSEA: Gene set enrichment analysis
IAA: Indole-3-acetic acid
IAAH: Indoleacetamide hydrolase
IBD: Inflammatory bowel disease
ICA: Indole-3-carbaldehyde
ID: Indolepyruvate decarboxylase
IDO: Indoleamine 2,3-dioxygenases
IL: Interleukin
ILA: Indole-3-lactic acid
ILD: Indolelactate dehydrogenase
IPA: Indole-3-propionic acid
KEGG: Kyoto Encyclopedia of Genes and Genomes
KO: Knockout
LEfSe: Linear discriminant analysis effect size
*L. reuteri*: *Lactobacillus reuteri*
MLNs: Mesenteric lymph nodes
PBS: Phosphate-buffered saline
PCR: Polymerase chain reaction
PXR: Pregnane X receptor
RNA-seq: RNA sequencing
rRNA: Ribosomal RNA
Th: T helper
UC: Ulcerative colitis
WT: Wild-type
*C. sporogenes*: *Clostridium sporogenes*
