# Supplementary material for: Microbiota-derived indoles alleviate intestinal inflammation and modulate microbiome by microbial cross-feeding
Source: Microbiome. 2024 Mar 19;12:59. doi: 10.1186/s40168-024-01750-y (PMC10949743; doi:10.1186/s40168-024-01750-y)

**Supplementary figures**

**Figure S1. *L. reuteri* protects mice from DSS-induced colitis.** (A) Experimental scheme of a colitis-recovery-colitis-colonic microbiota transplantation (CMT) model (n = 18-20). Six-week-old C57BL/6 mice were orally gavaged with 200 µl of PBS with or without *L. reuteri* I5007 (10^9^ CFU/mL) daily for three weeks, followed by a week of 3% DSS administration in drinking water to induce colitis and then a 12-day self-recovery period. From day 40-47, a second round of colitis was induced with 2% DSS in drinking water for a week, followed by reciprocal CMT. The mice in the DSS group were gavaged with day-28 colonic bacteria from the DSS_I5007 group, while the mice in the DSS_I5007 group were administered with day-28 colonic microbiota from the DSS group. (B) Dynamic changes in the disease activity index (DAI) score from day 21 to 40. (C) Colonic histological scores of mice (n = 6). (D) Representative images of hematoxylin and eosin staining of the colonic sections. (E) PCoA analysis of the Bray–Curtis distances of the colonic microbiota among three groups of mice on day 40. (F) The α-diversity (Richness and Shannon indices) of the colonic microbiota (n=8) on day 40. The median value of each group is shown. ****p<0.0001, ***p<0.001, **p<0.01, *p<0.05; ns, not significant.


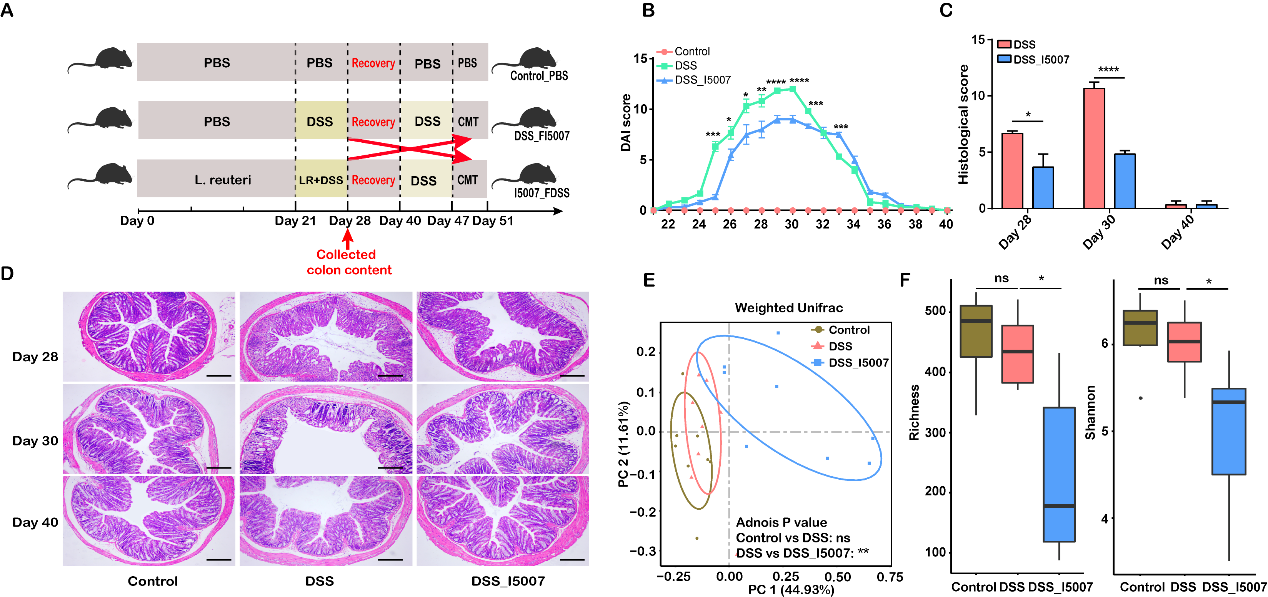


**Figure S2. The colonic microbiota of *L. reuteri*-supplemented mice protects mice from DSS-induced colitis.** Trial design saw the Supplementary Figure 1A legend for experimental details. The survival (A) and body weight changes (B) of mice (n = 5) were recorded between day 47-51. Statistical analysis was performed with weight changes using one-way ANOVA and Tukey’s post-hoc test. ^**^p < 0.01 indicates significant differences between DSS_FI5007 and I5007_FDSS groups on days 47 and 48. The histological score (C) and representative images (D) of hematoxylin and eosin-stained colonic sections of different groups of mice on day 51 (n = 4). (E) The colon lengths of different groups of mice on day 51 (n = 4). Total Th17A cell numbers (F) and Treg cell numbers (G) in the common lymphoid progenitor (CLP) cells as well as Th17A cell numbers (H) and Treg cell numbers (I) in mesenteric lymph nodes (MLNs) were determined by flow cytometry (n = 4). ^*^p < 0.05, ^**^p < 0.01, ^***^p < 0.001, ^****^p < 0.0001; ns, not significant.


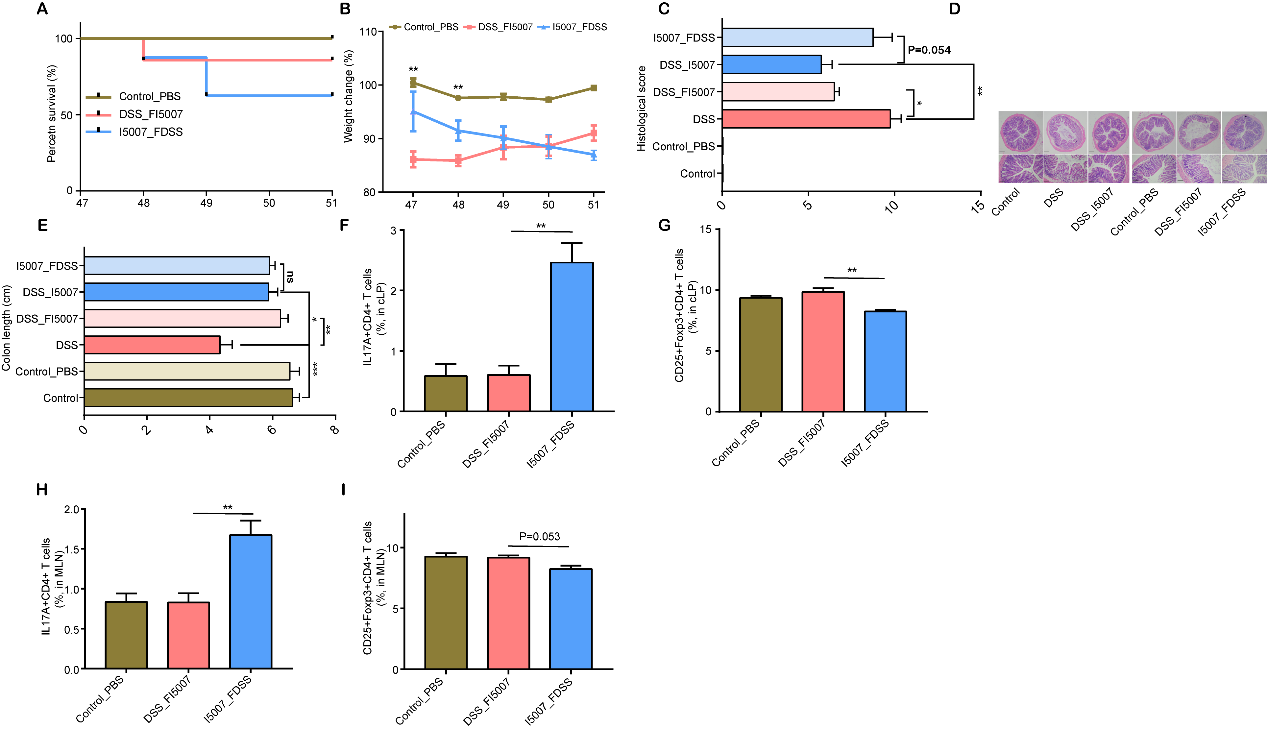


**Figure S3. *L. reuteri* affects the levels of tryptophan metabolites and the expression of AhR-related genes.** Trial design saw the Supplementary Figure 1A legend for experimental details. (A) Serum concentrations of tryptophan metabolites (kynurenine, tryptophan, 5-HT, and ILA) (n = 10) on day 28 at the end of the trial. (B) Scatter plot of Kyoto Encyclopedia of Genes and Genomes (KEGG) pathway enrichment results of differentially expressed genes. The rich factor indicates the number of differentially expressed genes located in the KEGG pathway. The color represents the significance of the difference. (C) Gene set enrichment analysis (GSEA) results show negative enrichment of inflammation-related gene sets, which were changed after *L. reuteri* treatment compared with the DSS group. (D) Changes in AhR and its target genes (*Cyp1a1* and *IL-22*) in the colon of mice (n = 6). (E) Principal component analysis (PCA) plot of functional profiles with gene modules. (F) Correlation network analysis between differentially expressed genes and bacteria based on Spearman’s correlation. Red connections indicate a positive correlation (r > 0.4, FDR < 0.05) and blue connections represent negative correlations (r < -0.4, FDR < 0.05), while gray connections indicate no correlation (FDR > 0.05). ^*^p < 0.05, ^**^p < 0.01, ^***^p < 0.001, ^****^p < 0.0001; ns, not significant.


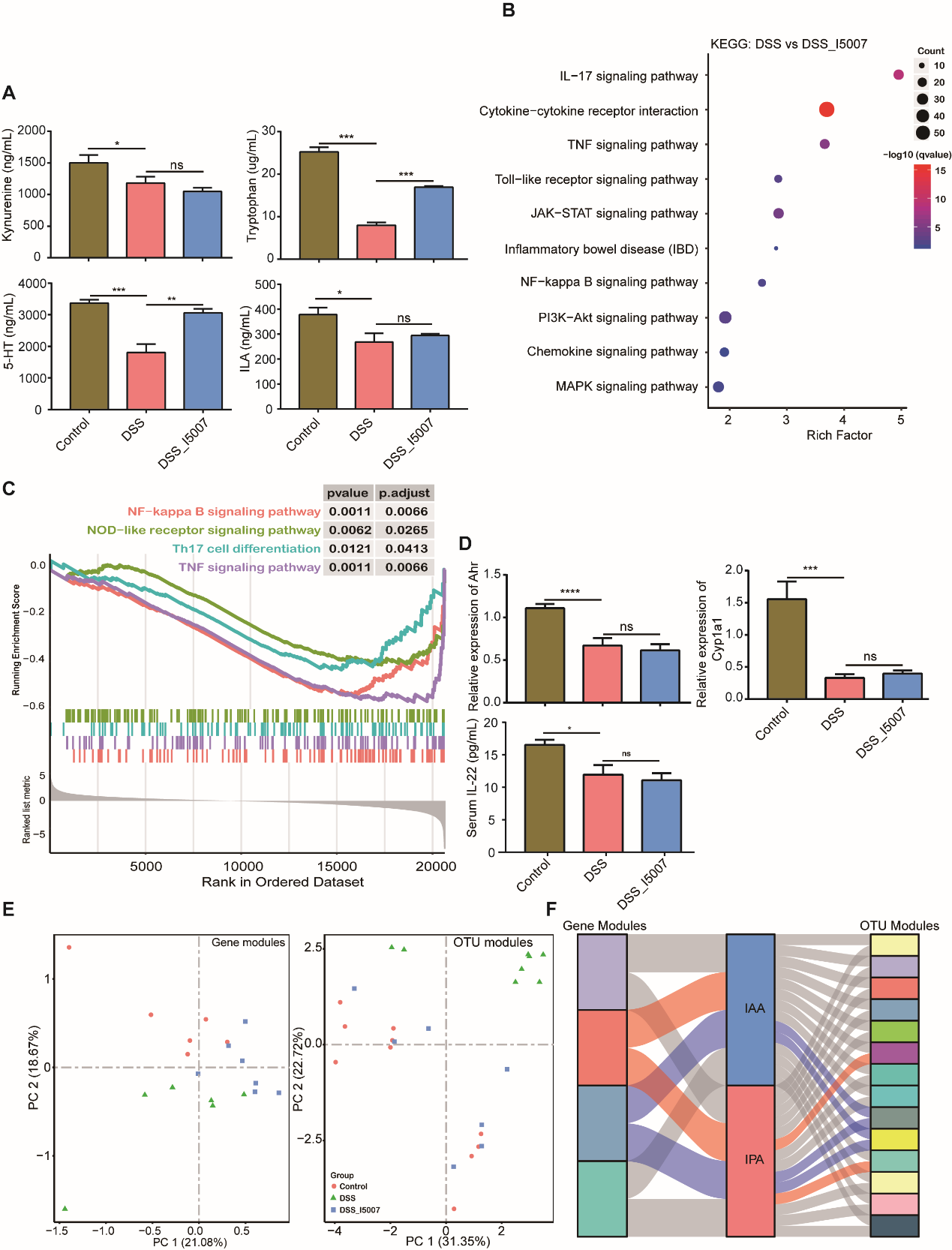


**Figure S4. IPA and IAA ameliorate DSS-induced colitis in mice.** (A) Trial design (n = 5). Colitis was induced by providing 3% DSS in the drinking water of mice for 7 days with or without oral administration of 20 or 40 mg/kg IPA or IAA. (B) Body weight changes during the trial. (C) The colon length of mice among different groups of mice on day 7. (D) Histological score (upper panel) and representative images of hematoxylin and eosin staining (lower panel) of the colonic sections on day 7. Scale bars represent 50 µm (n = 5). (E) Goblet cell changes in the colon of mice (n = 3). The upper panel shows the goblet cells per crypt of the colon, and the lower panel shows representative images of alcian blue staining for goblet cells in the inner mucus layer of colonic sections. (F) Levels of IL-1β and TNF-α mRNAs in the colon (n = 5) assessed using RT–qPCR. (G) Expression of the occludin and E-cadherin proteins (n = 3) determined by Western blotting. (H) Colonic expression of the *AhR*, *Cyp1a1* and *Pxr* mRNAs (n = 3) by RT-qPCR. ^*^p < 0.05, ^**^p < 0.01, ^***^p < 0.001, ^****^p < 0.0001; ns, not significant.


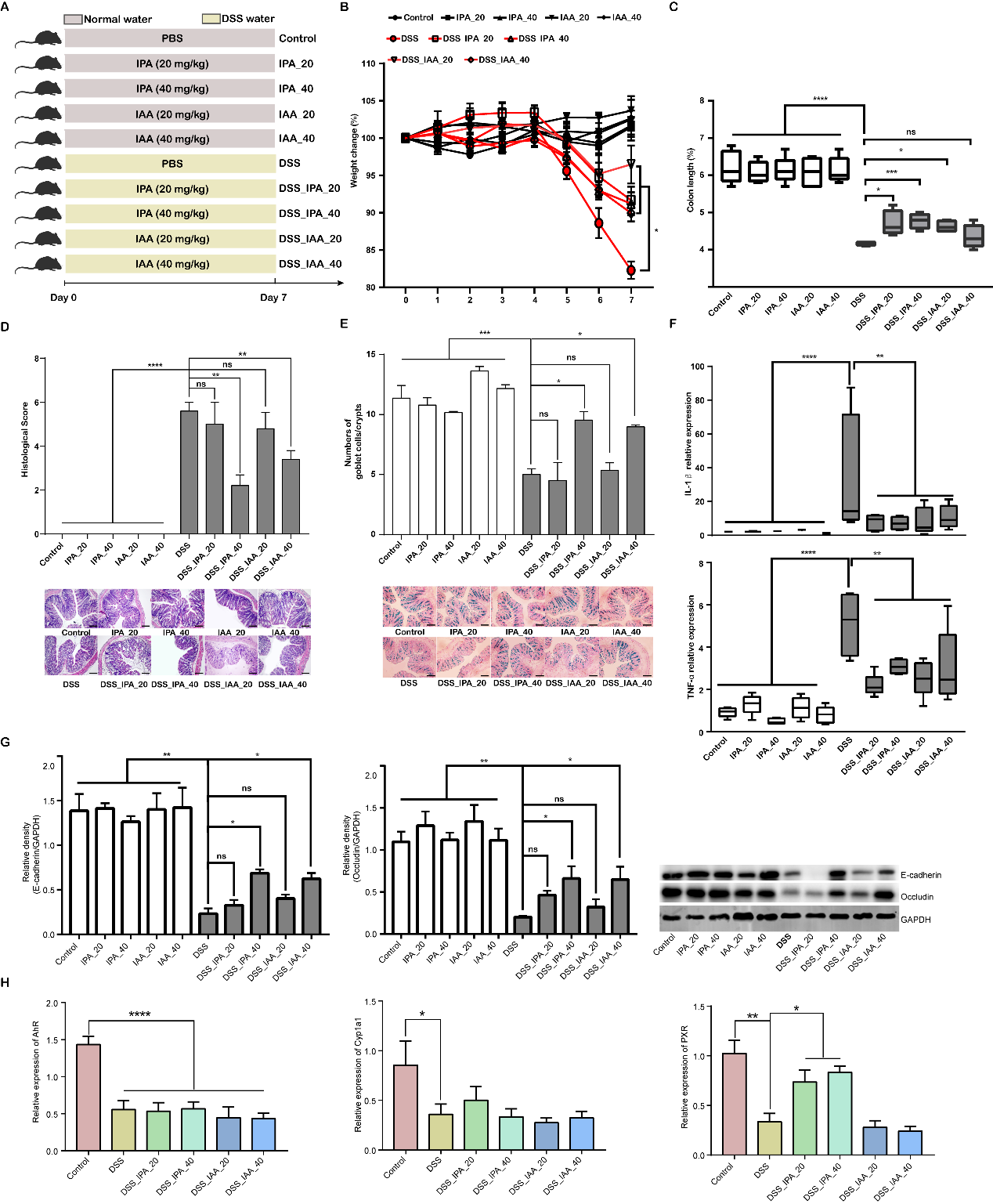


**Figure S5. IPA and IAA induce a shift in the colonic microbiota composition of mice.** Colitis was induced by providing 3% DSS in the drinking water of mice for 7 days with or without oral administration of 20 or 40 mg/kg IPA or IAA. (A) Boxplots of α-diversity (richness, Simpson, and Shannon indices) of the colonic microbiota on day 7 (n = 4). The medians of the data are shown. The statistical analysis was performed using the Wilcoxon rank-sum test. ^*^p < 0.05; ns, not significant. (B) PCoA plot of the Bray–Curtis distance to assess of β-diversity of the colonic microbiota on day 7 (n = 4) among different groups of mice.


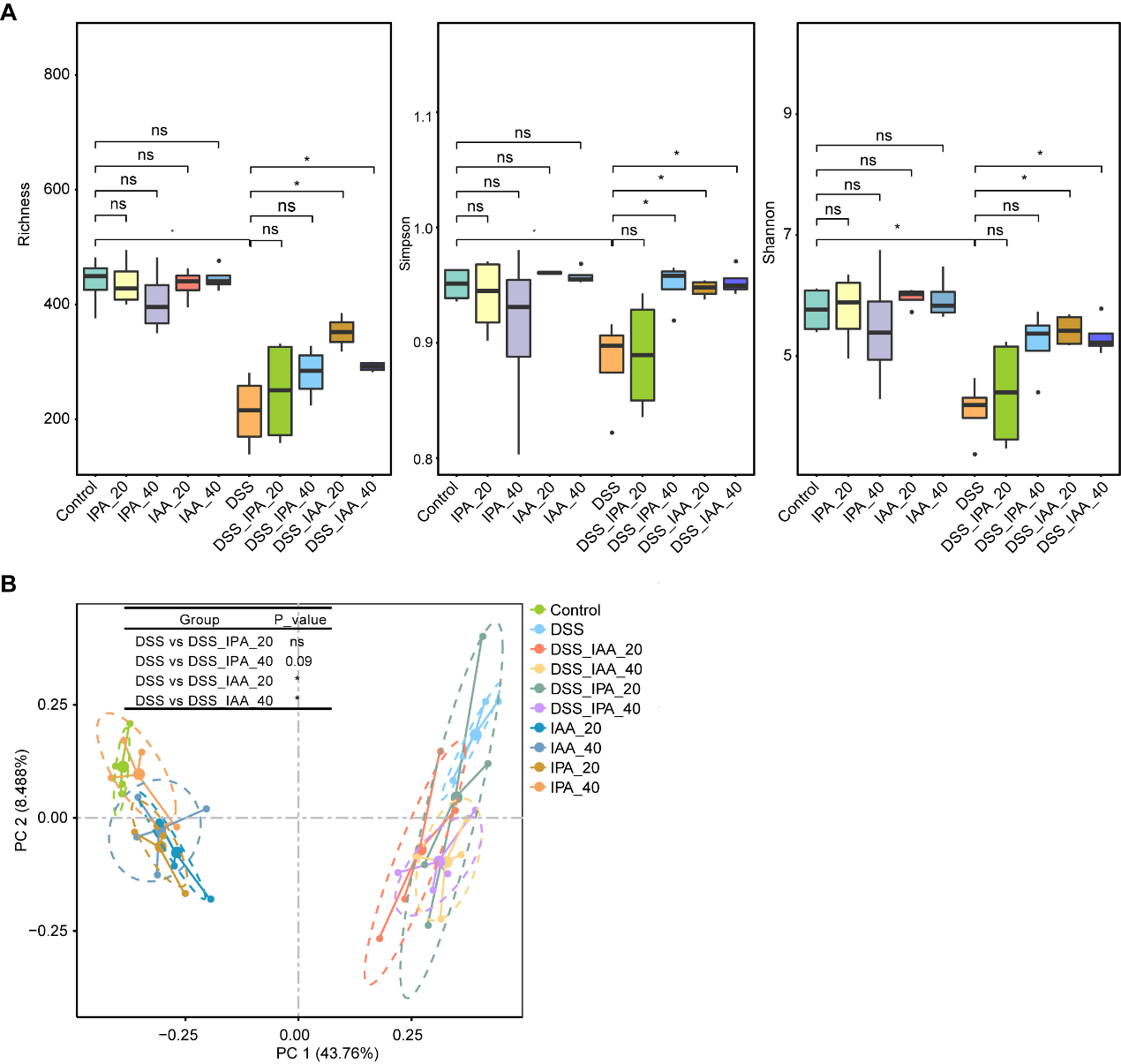


**Figure S6. Measurement of indole derivatives in the cell-free supernatant.** (A) Different *Lactobacillus* species were cultured under anaerobic conditions with an addition of 10% de Man Rogosa Sharpe (MRS) medium for 20 h at 37°C, followed by measurement of four different indole derivatives in the supernatant of each anaerobic culture. (B) *L. reuteri* I5007 was incubated with different concentrations of tryptophan under anaerobic conditions for 20 h at 37°C, followed by measurement of ILA in the supernatant.


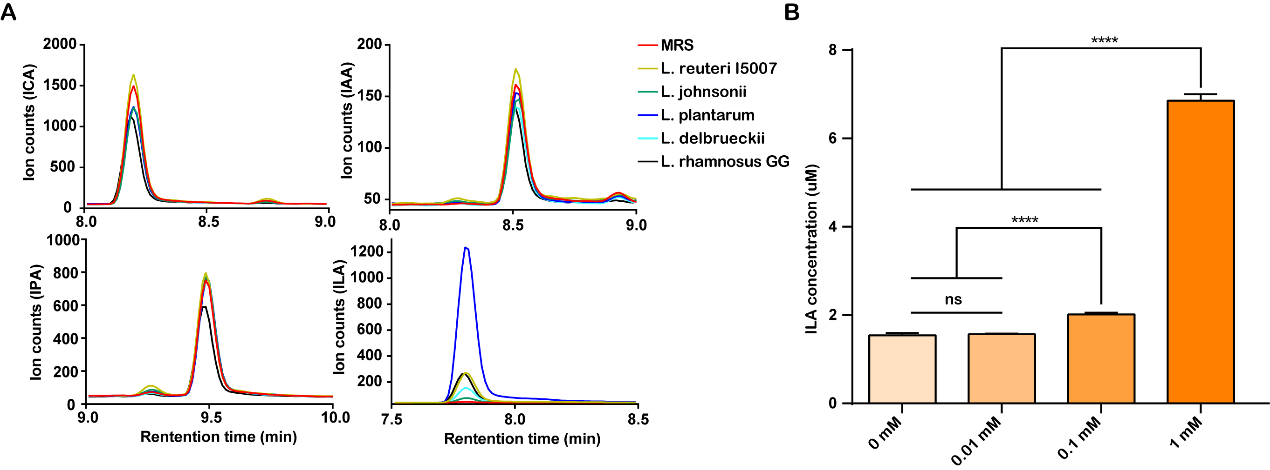


**Figure S7. ILA alleviates DSS-induced colitis and alters the intestinal microbiota composition.** Six-week-old C57BL/6 mice were orally gavaged with 200 µl of PBS with or without 20 mg/kg ILA daily for one week. Colitis was induced by adding 3% DSS in drinking water. (A) Body weight changes among three groups of mice (n = 6). (B) The colon length of mice among three groups of mice (n = 6). (C) Levels of TNF-α and IL-1β in the serum (n = 3). (D) Histological scores of the colon (n = 6). (E) Representative images of hematoxylin and eosin staining of the colonic sections. (F) Richness and Shannon index of the colonic microbiota among three groups of mice. (G) PCoA plot of the Bray–Curtis distance among three groups of mice. (H) Relative abundance of top 15 genera. *p<0.05, **p<0.01, ***p<0.001, and ****p<0.0001.


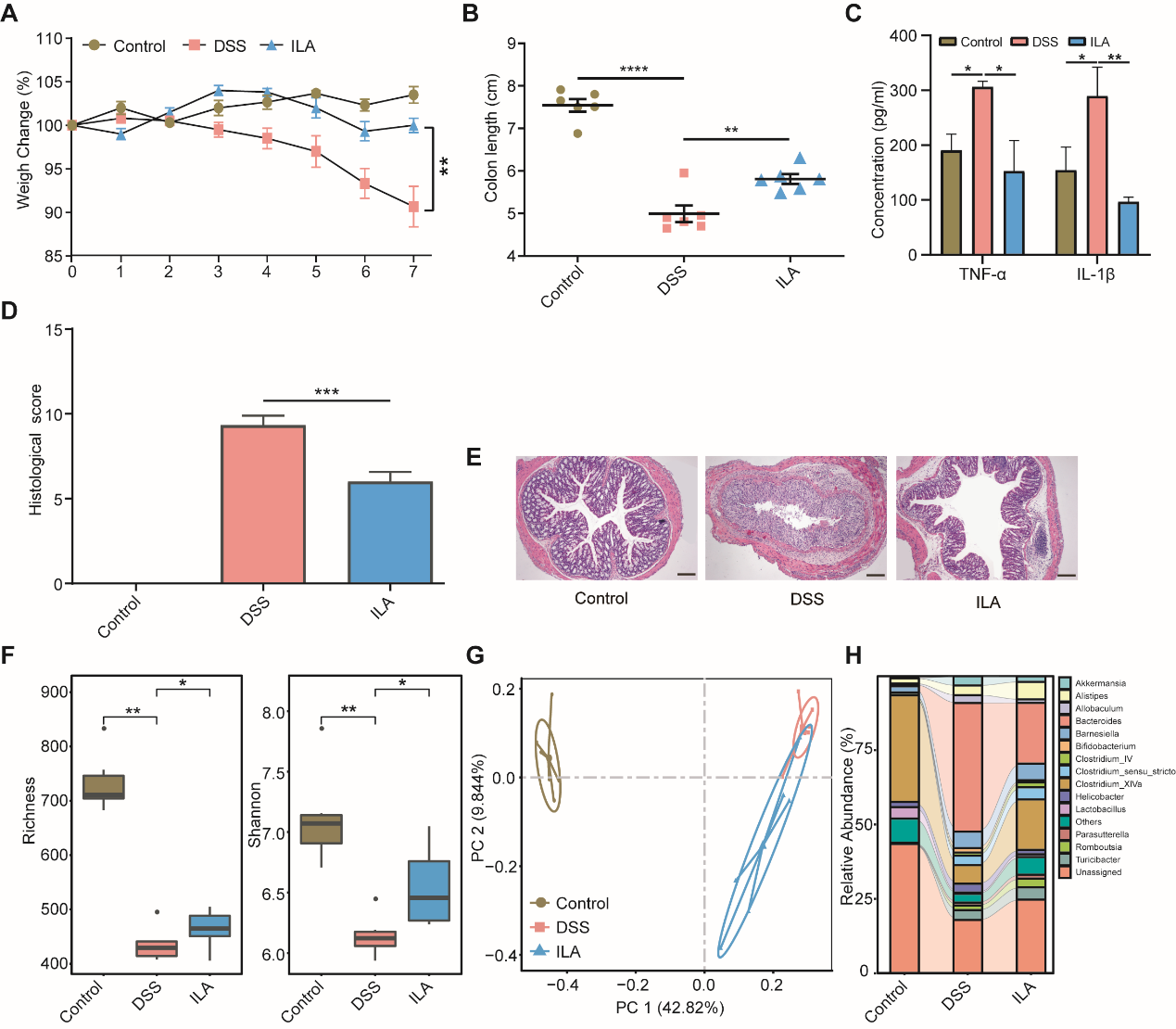


**Figure S8. ILA alleviates alters the colonic microbiota composition of DSS-treated mice.** (A) Trial design. The intestinal microbiota of 6-week-old C57BL/6 mice (n = 5) was depleted with a cocktail of antibiotics in drinking water for two weeks, followed by 3% DSS in drinking water with or without oral gavage of 20 mg/kg ILA daily for another week. (B) Weight changes between day 14-21. (C) The colon length of mice among different groups. Histological score (D) and representative images of hematoxylin and eosin staining of the colonic sections (E) were shown. (F) Trial design. Mice (n = 5) were orally administered with or without 20 mg/kg ILA for a week, followed by induction of colitis with 3% DSS in drinking water for another week with or without oral supplementation of 20 mg/kg ILA or CH223191. (G) Body weight changes between day 7-14 among different groups of mice. (H) The colon length of mice on day 14 in different groups. Histological score (I) and representative images of hematoxylin and eosin staining of the colonic sections (J) were shown. ^*^p < 0.05, ^**^p < 0.01, and ^****^p < 0.0001, ns, not significant.


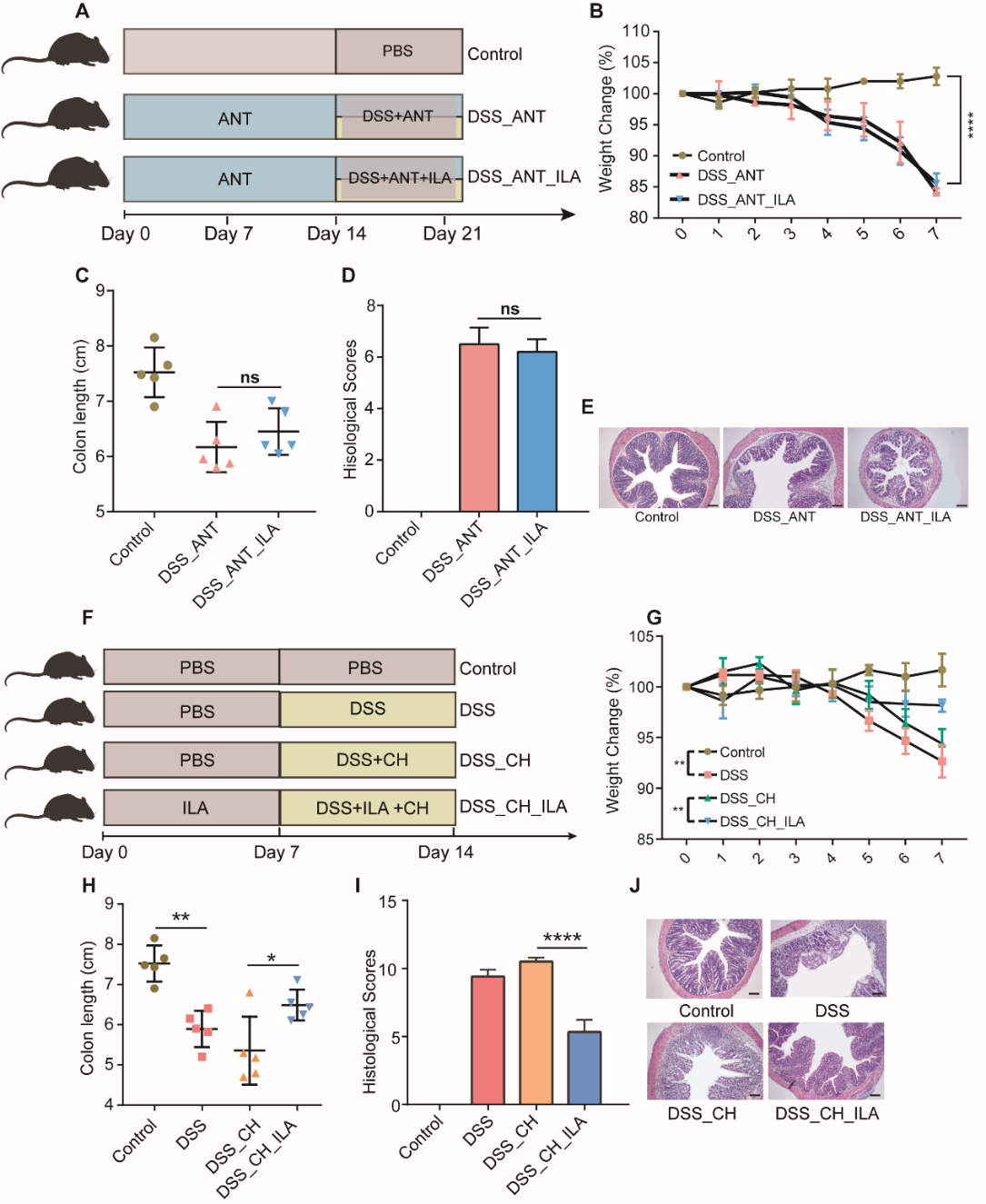


**Figure S9. Bacterial and bacterial enzymes involved in microbial metabolism of tryptophan are altered in IBD patients.** Human HMP2 metatranscriptomic sequencing reads of 46 Crohn's disease (CD) patients, 21 ulcerative colitis (UC) patients, and 11 healthy controls (nonIBD) were retrieved from the Inflammatory Bowel Disease Multiomics Database. (A) PCoA plot of the Bray–Curtis distance along CD, UC, and healthy patients. (B) Normalized abundances of acyl-CoA dehydrogenase (ACD) among CD, UC, and healthy patients. Statistical significance was determined from linear mixed effects models. *p < 0.05. (C) Distribution of ACD in the top 10 genera (C) and species (D). The changes of top 10 ACD-expressing bacterial genera (E) and species (F) among CD, UC, and healthy patients.


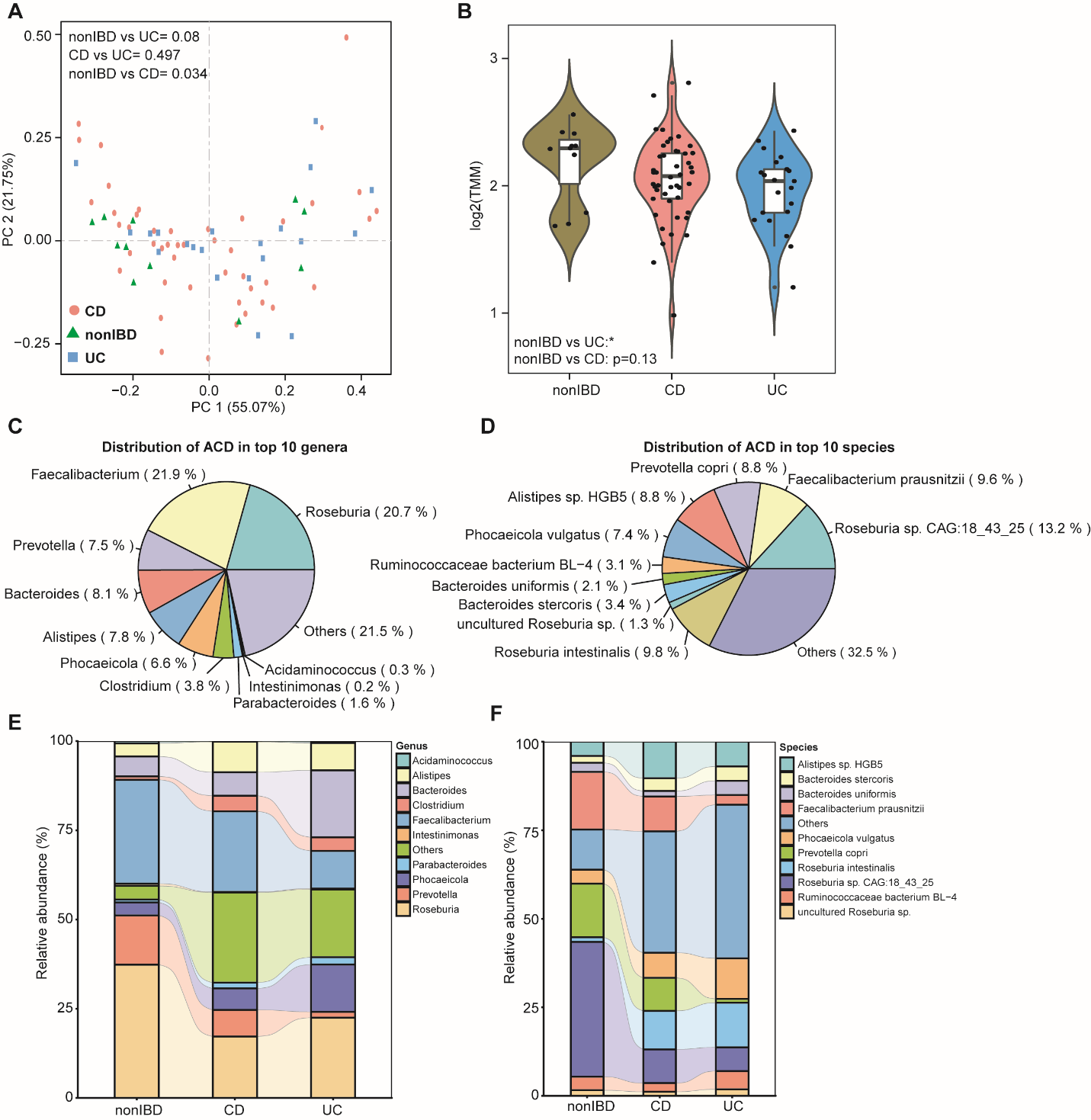

Supplement: Supplementary file 2 — Additional file 1: Figure S1. L. reuteri protects mice from DSS-induced colitis. (A) Experimental scheme of a colitis-recovery-colitis-colonic microbiota transplantation (CMT) model (n = 18-20). Six-week-old C57BL/6 mice were orally gavaged with 200 µl of PBS with or without L. reuteri I5007 (109 CFU/mL) daily for three weeks, followed by a week of 3% DSS administration in drinking water to induce colitis and then a 12-day self-recovery period. From day 40-47, a second round of colitis was induced with 2% DSS in drinking water for a week, followed by reciprocal CMT. The mice in the DSS group were gavaged with day-28 colonic bacteria from the DSS_I5007 group, while the mice in the DSS_I5007 group were administered with day-28 colonic microbiota from the DSS group. (B) Dynamic changes in the disease activity index (DAI) score from day 21 to 40. (C) Colonic histological scores of mice (n = 6). (D) Representative images of hematoxylin and eosin staining of the colonic sections. (E) PCoA analysis of the Bray–Curtis distances of the colonic microbiota among three groups of mice on day 40. (F) The α-diversity (Richness and Shannon indices) of the colonic microbiota (n=8) on day 40. The median value of each group is shown. ****p<0.0001, ***p<0.001, **p<0.01, *p<0.05; ns, not significant. Figure S2. The colonic microbiota of L. reuteri-supplemented mice protects mice from DSS-induced colitis. Trial design saw the Supplementary Figure 1A legend for experimental details. The survival (A) and body weight changes (B) of mice (n = 5) were recorded between day 47-51. Statistical analysis was performed with weight changes using one-way ANOVA and Tukey’s post-hoc test. **p < 0.01 indicates significant differences between DSS_FI5007 and I5007_FDSS groups on days 47 and 48. The histological score (C) and representative images (D) of hematoxylin and eosin-stained colonic sections of different groups of mice on day 51 (n = 4). (E) The colon lengths of different groups of mice on day 51 [file 40168_2024_1750_MOESM1_ESM.docx]
